# Computational Investigation of GNMT-Catalyzed Methyl Transfer Reaction: Integrating MD, QM, and ML Approaches

**DOI:** 10.1002/jcc.70434

**Published:** 2026-06-15

**Authors:** Jonathan Epih, Anjali Arya, Saghar Gomrok, Qianyi Cheng

**Affiliations:** Department of Chemistry, University of Memphis, Memphis, Tennessee, USA

**Keywords:** enzyme catalysis, machine learning, methyl-transfer, molecular dynamics, QM-cluster modeling

## Abstract

The glycine N-methyltransferase (GNMT) reaction was examined using an integrated workflow combining molecular dynamics (MD), quantum mechanical (QM) cluster calculations, and machine learning (ML) analysis. Instead of relying on a single crystal-like conformation, multiple MD simulations were used to sample diverse reactant (SAM + glycine bound to GNMT) and product (SAH + sarcosine bound to GNMT) state geometries for QM cluster modeling. Across more than 150 QM-cluster models constructed by the Residue Interaction Network ResidUe Selector (RINRUS) from selected MD frames with and without explicit waters, the computed activation and reaction free energies span broad ranges (7 to 25 kcal mol^−1^ and −36 to +3 kcal mol^−1^), demonstrating a strong dependence on the initial MD conformation. Product-state consistently yields lower reaction barriers, while explicit water introduces only small shifts in energetics and preserves the relative ordering among frames. The two-coordinate potential energy surface (PES) offers only limited insight and cannot fully account for the observed energetic variability. These QM-cluster models were further analyzed using machine-learning methods to identify structural descriptors that correlate with the observed energy variations and provide insight into their structural origin. ML models trained on multiple feature representations show that the donor–methyl–acceptor distances are the most informative and yield the strongest predictive accuracy, while higher dimensional solvent- or residue-based features contribute comparatively little. Overall, the results highlight the importance of conformational sampling for reliable QM-cluster energetics and point toward more expressive structure-to-property representations for analyzing enzymatic reactions.

## 1 | Introduction

Enzymes are among the most effective catalysts known in nature, capable of accelerating chemical reactions by many orders of magnitude under physiological conditions [1, 2]. Their ability to stabilize transition states and direct reactivity with high specificity underlies a wide range of biological processes, from metabolism to signaling [3]. Understanding the molecular principles of enzyme function has therefore been a long-standing goal in biochemistry and molecular biology, both for its fundamental importance and its potential to guide applications in drug design and biotechnology [4]. From a computational perspective, however, studying enzymatic processes remains particularly challenging. Identifying the mechanistic steps is often tractable, but obtaining reliable energetics that meaningfully correspond to experimental measurements requires careful treatment of conformational variability and local interactions within the active site [5, 6].

To address these challenges, a variety of computational methods have been applied to the study of enzyme catalysis. Classical molecular dynamics (MD) simulations are widely used to capture protein flexibility and conformational sampling [7, 8], while hybrid quantum mechanics/molecular mechanics (QM/MM) approaches provide a rigorous framework for modeling chemical transformations within the enzyme environment [9–12]. More approximate schemes, such as semiempirical quantum methods, offer additional ways to study reaction pathways at reduced computational cost, albeit with lower accuracy [13, 14].

The QM cluster approach, where only the subset of residues and atoms surrounding the active site that contribute directly or indirectly to the reaction mechanism are modeled quantum mechanically, offers a practical way to study enzymatic reaction mechanisms at high electronic structure accuracy [15, 16]. However, energies obtained from cluster models can be highly sensitive to choices made during model construction, including the size of the QM region and the selection of residues and atoms to include [17–19]. Beyond composition, the initial conformation of the cluster is equally critical. Even small differences in the MD-derived starting geometries can lead to substantial variations in the QM optimized reactant, transition state, and product energies. This sensitivity arises because QM energies depend strongly on subtle geometric differences: small geometric perturbations across many atoms can alter noncovalent interactions, polarization, and electronic structure, collectively producing large shifts in the computed barriers and reaction free energies [20]. This sensitivity of computed energetics to structural variations has also been highlighted in studies of QM/MM free-energy convergence [21].

**SCHEME 1 |:**
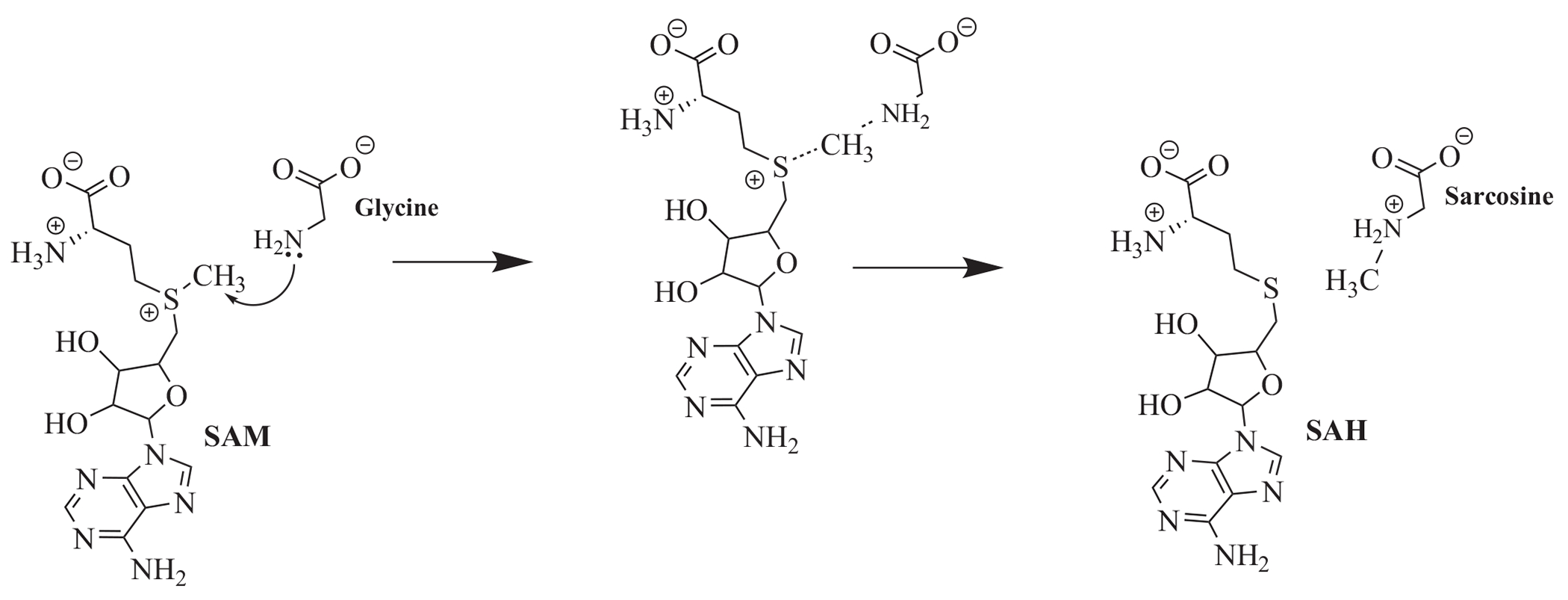
Reaction mechanism for GNMT-catalyzed methyl transfer. Glycine accepts a methyl group from S-adenosyl-L-methionine (SAM) to form sarcosine and S-adenosyl-L-homocysteine (SAH).

In this study, we systematically investigate how conformational variability in MD-derived starting structures and residue composition of the QM regions influence the computed energetics of an enzymatic methyl-transfer reaction. We focus on glycine N-methyltransferase (GNMT), a well characterized system experimentally and computationally. GNMT catalyzes the transfer of a methyl group from S-adenosyl-L-methionine (SAM) to glycine, to produce sarcosine and S-adenosyl-L-homocysteine (SAH) (Scheme 1). The enzyme functions as a homotetramer, with each subunit containing an independent active site, and plays a central role in regulating cellular one-carbon metabolism by linking the folate and methionine cycles [22]. Experimental measurements of GNMT’s kinetic parameters (turnover rates of 27.0 min ^−1^ at 30°C and 174.5 ± 23.2 min ^−1^ at 37°C) correspond to activation free energies of approximately 18.2 and 17.4–17.6 kcal mol^−1^, respectively [22, 23]. A prior QM-cluster study by Cheng and DeYonker has also established a computational benchmark for GNMT [18]. Together, this catalytic mechanism, its experimentally established energetics, and the availability of prior QM-cluster models make GNMT an ideal system.

To systematically evaluate the impact of structural variability, MD simulations were employed to sample the conformational ensemble of the GNMT enzyme-ligand complex, from which representative structures were selected as starting geometries for QM-cluster calculations. This approach allows us to compare the activation and reaction energies obtained from these models and directly assess how differences in initial geometry influence computed energetics.

Beyond quantifying how initial geometry affects QM results, we further sought to identify which structural features of the enzyme-ligand complex derived from the starting conformations most strongly correlate with the computed activation and reaction energies. To accomplish this, statistical and classical machine-learning (ML) methods were employed to evaluate multiple representations of enzyme conformation and to assess their predictive value for activation and reaction energies. These analyses provide a systematic framework for interpreting how conformational variability propagates into QM-cluster modeling outcomes.

## 2 | Methods

### 2.1 | Initial Structure Preparation

In this study, the X-ray crystal structure of wild-type GNMT (PDB ID: 1NBH) [22] was used as the starting structure, the same structure employed in previous work by Cheng and DeYonker. In this crystal structure, each subunit of the homotetramer is bound to S-adenosyl-L-methionine (SAM) and acetate (ACT), a competitive inhibitor occupying the substrate-binding pocket. To model the methyl transfer reaction, we used the chain A subunit and replaced ACT with the native substrate glycine. Notably, the crystal structure captures the enzyme in its reactant state, where the sulfur atom of SAM (SD) remains covalently bonded to the carbon atom of the methyl group (CE). To generate a corresponding product-state model, the methyl group (CH_3_) was repositioned from SAM to the amine nitrogen atom (N) of glycine to form a covalent N-CE bond, yielding the product-state geometry, while leaving the overall enzyme conformation unchanged.

### 2.2 | Molecular Dynamics Simulations

Molecular dynamics (MD) simulations were then performed on both the reactant- and product-state structures to examine conformational dynamics before and after methyl transfer. The required topology and coordinate files were generated using the AMBER 18 suite of programs [24]. Hydrogen atoms were added using the reduce program [25], and force field parameters were assigned accordingly, with the substrates (glycine/sarcosine) and cofactors (SAM/SAH) parameterized using the General Amber Force Field (GAFF) [26], and the protein residues treated with the ff14SB force field [27]. Each complex was solvated in a TIP3P explicit solvent box with a 15 Å solvent buffer [28], and the system was neutralized by adding two chloride ions (Cl^−^).

Energy minimization of each solvated system was carried out with a 2.0 kcal mol^−1^ Å^−2^ positional restraints applied to all heavy atoms to preserve the overall structure of the protein-ligand complex. The restrained systems were then heated from 0 to 300 K under NVT conditions using a Langevin thermostat (collision frequency 2.0 ps^−1^). SHAKE constraints were applied to all bonds involving hydrogen atoms [29], and long-range electrostatics were treated using the particle-mesh Ewald (PME) method [30, 31] with an 8.0 Å cutoff for nonbonded interactions.

Heating was followed by two sequential equilibration stages under NPT conditions at 300 K and 1 atm. The same positional restraints were applied to all heavy atoms in the first equilibration stage, and restraints were removed in the second stage to allow full relaxation of the system. Pressure was regulated using isotropic position scaling (relaxation times of 1.0 and 2.0 ps for restrained and unrestrained phases, respectively), and temperature was controlled with a Langevin thermostat.

Production MD simulations were performed for 300 ns under NPT conditions at 300 K and 1 atm with no positional restraints. SHAKE constraints were applied throughout to all bonds involving hydrogen. Thermodynamic quantities remained stable during the production phase, indicating that the systems were well equilibrated and suitable for structural analysis. This multi-stage simulation protocol was applied independently to four systems: two representing the reactant state and two representing the product state.

### 2.3 | Frame Selection

To identify representative structures for further analysis, three frame selection strategies were applied.

#### 2.3.1 | Early-Stage Frames

Four frames were obtained from the early-stage MD simulations by taking the average structures over the first 100 frames of each trajectory (two reactant-state simulations and two product-state simulations), following the same protocol used in MMPBSA analysis [32]. These averaged geometries closely resemble the X-ray crystal conformation and contain no explicit water molecules.

#### 2.3.2 | Clustering-Based Selection

Hierarchical agglomerative clustering was applied to the four independent MD trajectories, using the CPPTRAJ module of AMBER [33], based on the root-mean-square deviation (RMSD) of protein backbone heavy atoms (C, N, O, and C_α_). An average linkage algorithm with an epsilon cutoff of 3.0 Å was used to define 10 clusters per trajectory. The centroid structure of each cluster was then selected as the representative frame, yielding a total of 40 frames (20 reactant-state and 20 product-state).

#### 2.3.3 | Random Sampling

A second set of 40 frames was selected through random sampling from the same four MD trajectories. To ensure that this set remained distinct, any frames selected in the clustering analysis were excluded. Because not all MD-generated conformations have geometries suitable for the downstream QM calculations of the reaction mechanism, 40 candidate frames were initially sampled from the MD trajectory, and 35 frames from the 40 were selected in which the SAM sulfur, the methyl group, and the glycine amine remained properly oriented for methyl-transfer. The remaining five frames where water molecules or side chains obstructed the path connecting these atoms, or where the reactive groups were oriented too far from each other, were discarded.

PDB files for all MD frames selected by these procedures are provided in the Supporting Information.

### 2.4 | Quantum Mechanical (QM) Cluster Model Construction

QM cluster models based on the selected frames were constructed using the Residue Interaction Network ResidUe Selector (RINRUS) toolkit [17, 34, 35], which identifies residues in close spatial proximity to the glycine substrate or the SAM cofactor using Probe [36], capturing five categories of interactions: hydrogen bonds, close contacts, wide contacts, small overlaps, and large overlaps. This proximity-based selection ensures that all chemically relevant interactions within the active site are retained while keeping the overall system size suitable for QM calculations.

QM calculations were performed on models constructed from the three frame selection sets (4 early-stage frames, 40 clustering-based frames, and 35 randomly sampled frames). These frames were used to generate four distinct sets of models.

The first QM model set was constructed from the four early-stage MD frames. Each model contained the full set of residues identified by RINRUS and had an average size of 423 atoms. During the QM geometry optimizations, all residue C_α_ atoms and selected residue C_β_ atoms were held fixed. The residue compositions and the frozen atoms are listed in Table 1, following the convention used in the study by Cheng and DeYonker.

The second QM model set was constructed from the 40 frames selected using the hierarchical agglomerative clustering approach. These models were built by including all interacting residues identified by RINRUS but excluding the explicit water molecules (identified by RINRUS from the MD solvent box as interacting with SAM and glycine), resulting in QM-cluster models containing an average of 430 atoms. The third QM model set was also constructed from the same 40 clustered frames. These models were built by including the same set of RINRUS-identified residues used in the second QM model set (no explicit water models) and adding the explicit water molecules identified by RINRUS, yielding QM-cluster models containing an average of 442 atoms. On average, about four explicit water molecules were included in these models, with the number of waters ranging from 1 to 10 across the model set.

**FIGURE 1 |:**
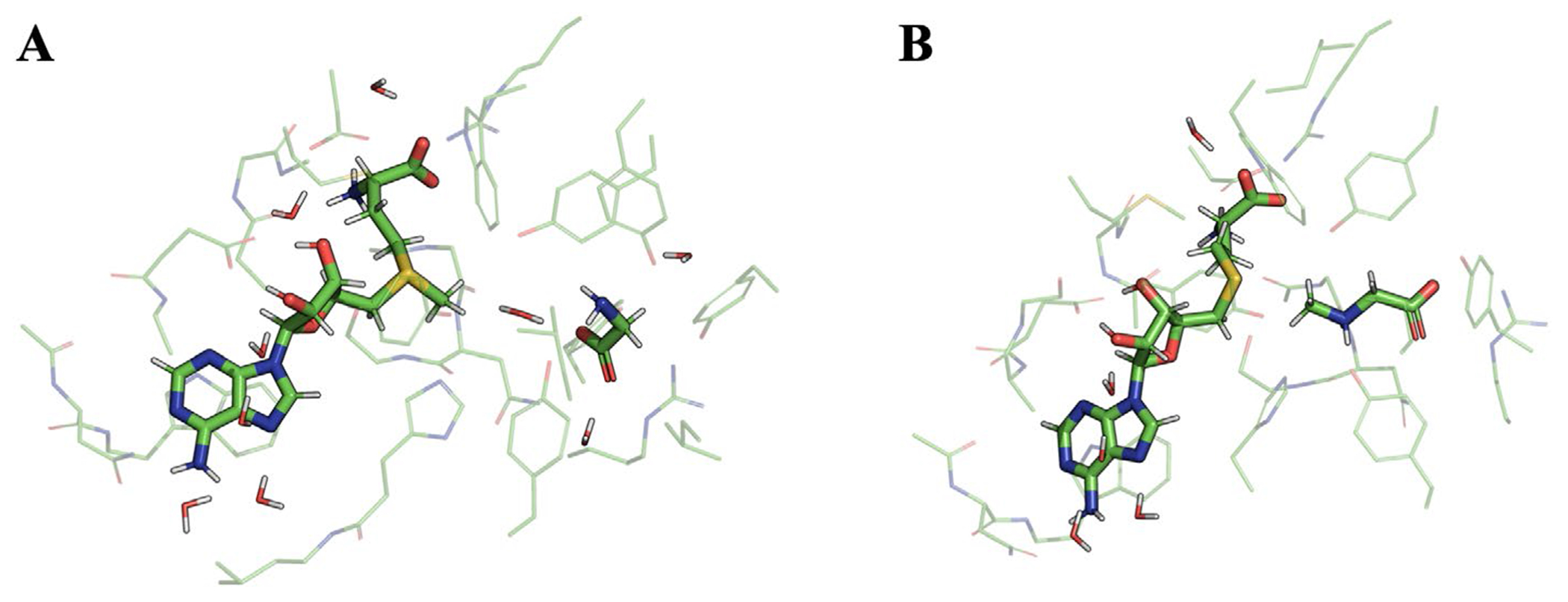
Representative QM-cluster models of the GNMT active site including explicit water molecules prior to QM optimization. (A) Reactant-state model constructed from an MD frame selected by hierarchical clustering. (B) Product-state model constructed from an MD frame selected by random sampling. The QM region includes the RINRUS-selected active-site residues surrounding SAM and glycine together with first-shell explicit water molecules identified by RINRUS.

Finally, the fourth QM model set was constructed from 35 randomly sampled MD frames. For each frame, two models were generated: one without explicit water and one with the explicit first-shell water molecules. The models without water contained an average of 423 atoms, while those with water contained an average of 441 atoms. These models contained an average of six explicit water molecules per cluster model (ranging from 3 to 9). In total, 154 QM-cluster models were constructed across all model sets. Representative QM-cluster models including explicit water molecules prior to QM optimization are shown in Figure 1.

The residue compositions and frozen atom information (all C_α_ atoms and selected C_β_ atoms) for the second through fourth QM model sets are provided in the Supporting Information (Tables S1–S4).

### 2.5 | Quantum Mechanical (QM) Calculations

All QM calculations were performed using the same computational settings as those employed by Cheng and DeYonker, using the Gaussian 16 software package [37]. Density functional theory (DFT) B3LYP exchange-correlation functional [38–42] was employed throughout with a mixed basis set scheme: 6-31G(*d*′) for S, O, and N atoms [43–45] and 6-31G for C and H atoms [46]. Empirical dispersion corrections were applied using Grimme’s D3 scheme with Becke-Johnson damping (GD3BJ) [47, 48] to improve the treatment of noncovalent interactions. Solvent effects were modeled using the conductor-like polarizable continuum model (CPCM) [49, 50] with a dielectric constant ϵ = 4.0. The CPCM implementation used Universal Force Field (UFF) atomic radii and a cavity scaling factor of *α* = 1.2. These computational settings were applied to all geometry optimizations and vibrational frequency calculations were carried out to identify minima and transition state (TS) structures on the potential energy surface. Each TS structure was used in intrinsic reaction coordinate (IRC) [51, 52] calculations, which confirmed their connectivity to the corresponding reactant and product geometries. Zero-point energies (ZPE) and thermal corrections to enthalpy and free energy were computed using unscaled vibrational frequencies at 1 atm and 310 K for the early-stage models, and at 1 atm and 298.15 K for all other cluster models.

Notably, for two QM-cluster models containing explicit water molecules that were built from the frames selected by hierarchical clustering, a transition-state structure corresponding to the methyl-transfer step could not be located: the TS optimization either failed to converge or converged to an unrelated geometry. These unsuccessful cases were excluded, resulting in only 38 of the 40 explicit-water models yielding valid TS structures.

PDB files of all optimized reactant, TS, and product structures are provided in the Supporting Information.

### 2.6 | Machine Learning Workflow

For the machine-learning analysis, the training and test datasets were defined according to the two MD frame selection strategies. All training datasets were constructed from the QM-cluster models and their corresponding MD frames which were derived from the hierarchical clustering procedure, while all test datasets were constructed from the QM-cluster models and MD frames derived from random sampling. For the explicit water QM-cluster models, only frames that yielded a converged TS structure were included. This resulted in 38 training data points and 35 test data points. For the models without explicit water, the entire set of 40 clustering selected frames were used for training. However, only 35 of the 40 randomly sampled frames were included in the test dataset to ensure that the test sets for the water and no-water models contained the same number of data points (Table 2). The computed Gibbs free energy of activation (Δ*G*^‡^) and reaction (Δ*G*_rxn_) of the QM-cluster models were used as regression targets.

To encode the enzyme’s initial conformation as captured in the MD frames, four distinct sets of feature representations were constructed. Each representation was generated using Python scripts and organized into a tabular format in which each row corresponded to a single frame or QM-cluster model and each column corresponded to an extracted structural feature. The detailed feature representations are shown in the Supporting Information (ML.xlsx file).

#### 2.6.1 | Donor–Methyl–Acceptor Distances Feature Representation

The first feature representation corresponded to the reaction coordinate for the methyl-transfer step and was defined using three key interatomic distances: the sulfur-nitrogen distance (SD-N), the carbon-nitrogen distance (CE-N), and the sulfurcarbon distance (SD-CE). The cartesian coordinates for these atoms were extracted from each MD frame, and the corresponding distances were computed for every structure.

#### 2.6.2 | Solvent–Derived Feature Representation

The second feature representation quantified the solvent environment by identifying and tracking the specific water molecules identified by RINRUS that directly interact with SAM and glycine in the MD-derived structures. This approach aimed to encode water molecules that were conserved or positioned near the substrate or cofactor, as these solvent interactions may influence the energetics of the methyl-transfer reaction. For each MD frame, solvent features were generated by counting, for each atom in SAM and glycine, how many of these explicit water molecules were found within hydrogen-bonding distance (3.5 Å). A water molecule contributed one count to a given SAM or glycine atom if any of its atoms (O, H1, or H2) fell within this cutoff; otherwise, it contributed zero. Each water molecule was counted at most once per SAM or glycine atom.

This counting procedure produced one solvent-based feature for each atom in SAM and glycine, yielding an initial set of 59 atom-specific descriptors. Columns that contained only zeros across all MD frames were removed. From the remaining features, mutual information (MI) analysis [53] was applied to rank their dependence on the target quantities, and the six most informative features (6 columns) were retained. MI measures the reduction in uncertainty about one variable (such as the energy) given knowledge of another (such as an interatomic or water-proximity descriptor), and it is able to capture both linear and nonlinear dependencies. In this context, MI was used as a univariate selection method, with each feature evaluated independently. This two-step filtering procedure reduced the dimensionality of the solvent feature space while preserving its most informative components.

#### 2.6.3 | Pairwise–Distance Representation

The third feature representation described the active-site geometry by quantifying pairwise distances between atoms belonging to residues located near the cofactor and substrate. This representation aimed to capture how the relative positioning of these residues varies across the MD-derived structures, thereby encoding conformational differences in the active-site environment.

To define a consistent set of residues for this representation, we first compiled the union of all residues that appeared in any of the QM-cluster models built using RINRUS, thereby establishing a uniform definition of the active-site subsystem across all MD frames and avoiding inconsistencies arising from model-to-model variations in residue selection. For each MD frame, pairwise distance features were then constructed by computing all interatomic distances between atoms within the active-site subsystem. This calculation included residue-residue, residue-SAM/glycine, and SAM-glycine pairs whose heavy-atom separation fell within a 10 Å cutoff.

Due to the high dimensionality of this pairwise representation, a two-stage feature selection process was applied to reduce the number of features and mitigate the risk of overfitting. The first stage involved grouping all atom-atom distances by the residue-residue pair they belonged to. Within each residue pair, the single atom–atom distance that showed the strongest relationship with Δ*G*^‡^ was identified using MI, thereby selecting one representative distance per residue pair. In the second stage, a random forest regressor (RFR) was trained on this reduced feature set. By leveraging the RFR’s ability to evaluate features jointly and incorporate nonlinear interactions [54], this step served as a powerful multivariate selection procedure. The feature-importance scores from the model were then used to identify the five most informative descriptors, effectively accounting for redundancies and interactions among the remaining distances.

#### 2.6.4 | Interaction–Type Representation

The final feature representation focused on non-covalent interactions of the active site subsystem, computed using the Probe software [36]. In this representation, only interactions between the active site residues and either SAM or glycine were evaluated. For each MD frame, Probe was run on the full structure to count the five categories of interactions. These five counts were then summed for each SAM and glycine atom to yield a single total interaction value per atom. This procedure generated a total of 59 interaction-type based features. To further reduce redundancy and identify the dominant modes of variation, principal component analysis (PCA) [55, 56] was applied to the 59 features. Retaining the components that together explained 81% of the total variance resulted in 16 principal components, which were then used as the final interaction-based descriptor set for subsequent modeling.

To evaluate the predictive power of these feature representations, several regression algorithms were applied to predict both the activation free energies (Δ*G*^‡^) and reaction free energies (Δ*G*_rxn_) obtained from the QM calculations. Given the limited dataset size, we employed classical machine learning methods due to their ease of interpretation, and effectiveness in small-sample settings. These included linear models (linear regression, ridge, lasso, elastic net) and nonlinear models (support vector regression, k-nearest neighbors, decision tree, random forest, AdaBoost, and gradient boosting). All models were implemented using the scikit-learn library [57] with default hyperparameters.

Model assessment was carried out for both datasets, with and without explicit solvent molecules, in two complementary ways. First, leave-one-out cross-validation (LOOCV) was performed on the corresponding training sets to obtain an internal estimate of model performance. LOOCV was used only as an error estimate and was not used for feature selection or any form of model tuning. Second, and more importantly, all trained models were evaluated on their respective independent test sets.

To establish a baseline for predictive performance, a dummy regressor was used that ignores structural features and simply predicts the mean of the training values for either Δ*G*^‡^ or Δ*G*_rxn_. For each model and feature representation, predictions on the test set were compared against the corresponding QM-derived energies. The primary evaluation metric was the mean absolute error (MAE), which quantifies the average deviation between predicted and true values. MAE values were computed for both energy types and for all combinations of models and feature representations. Performance that exceeded the dummy baseline indicated that the structural features contributed predictive value beyond random chance.

## 3 | Results and Discussion

**FIGURE 2 |:**
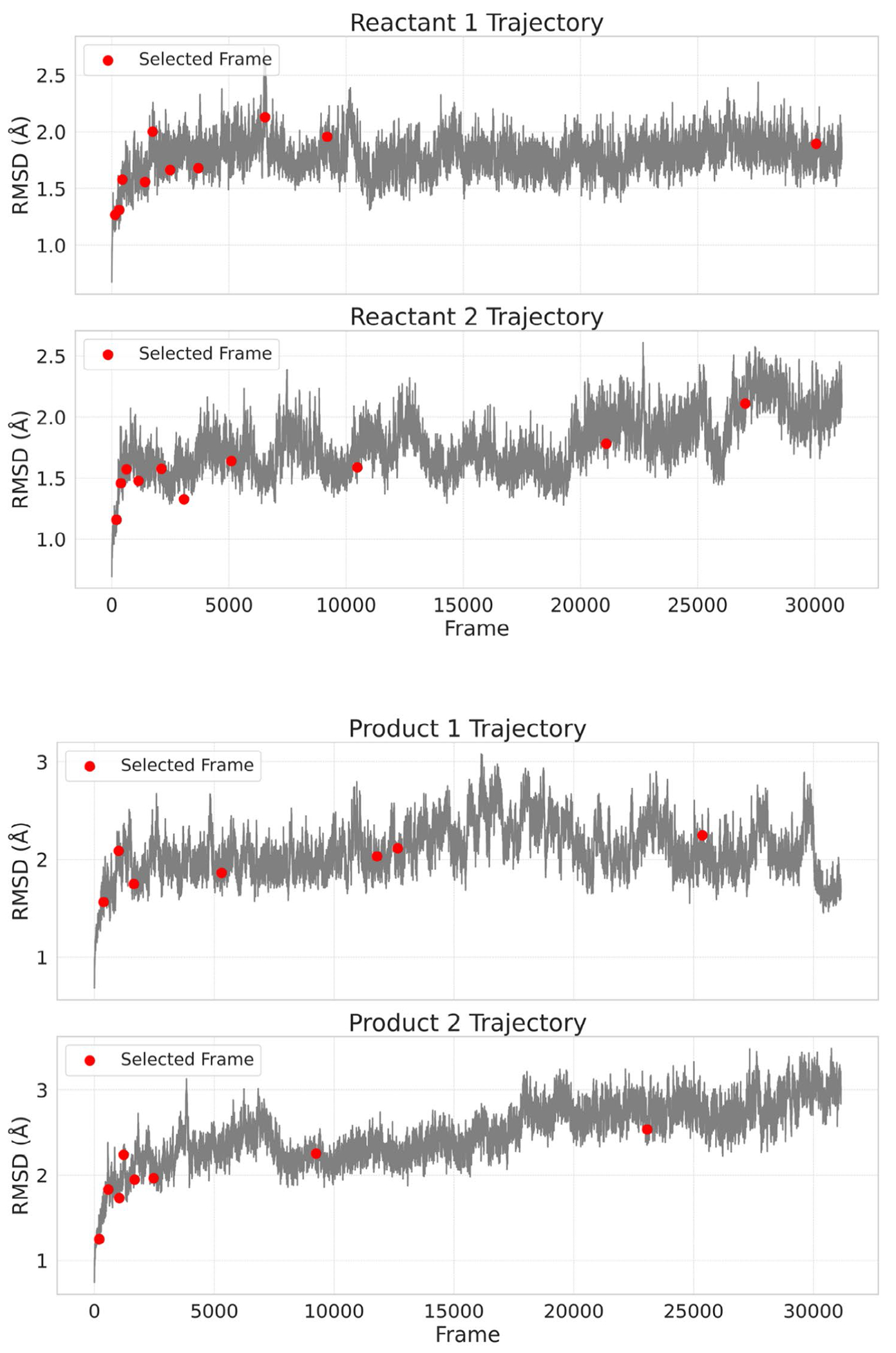
Backbone RMSD (N, C_α_, C, and O atoms) relative to the starting structure is shown for each trajectory. Frames selected for QM modeling are highlighted in red.

Before analyzing the QM-cluster models, we first evaluated the stability and conformational behavior of the MD simulations used to generate starting structures. Backbone RMSD relative to the X-ray crystal structure (Figure 2) shows stable fluctuations after equilibration, indicating that the simulation remained well behaved and sampled conformational space beyond the initial geometry. It should be noted that the first 16 residues, which form a flexible loop that undergoes large conformational changes, were excluded only from the RMSD plot shown in Figure 2. The RMSD analysis of the full enzyme is provided in Supporting Information (Figure S1).

### 3.1 | Early Stage QM-Cluster Models

The four QM-cluster models constructed from early stage MD frames (models A and B are from the reactant state frames, model C and D are from the product state frames) were examined first. As shown in Table 1, the four MD-derived frames exhibit modest deviations relative to the X-ray crystal structure, with backbone RMSDs ranging from 2.3 to 2.8 Å. Nevertheless, these small structural differences in the starting frames lead to notable differences in QM-computed energies. All four models yield elevated activation free energies (13.8 to 16.5 kcal mol^−1^) compared to the maximal model derived from the X-ray structure (9.1 kcal mol^−1^ [18], which is significantly lower than the experimental value of 17.5 kcal mol^−1^ [23]). The reaction free energies of the four models are more widely distributed (−29.7 to −15.8 kcal mol^−1^). Model A has a −29.7 kcal mol^−1^ free energy of reaction, which is comparable to the crystal reference. This suggests that the model preserves a large number of stabilizing interactions, consistent with the substantial overlap in residue composition contributing to the active-site environment. Models B, C, and D have higher reaction free energies, and model C shows the most pronounced destabilization of the product state. These differences likely reflect subtle conformational changes in active-site geometry that affect the relative stabilization of the product state along the reaction coordinate. Furthermore, the crystal structure-based model from the previous study contained 27 residues, 22 of which are conserved across all four MD derived models. These shared residues form a core set of interactions with SAM and glycine. The similarity in size and residue composition between the models suggests that the energetic variations likely stem from conformational changes introduced during the simulations, rather than differences in residue selection. Our results illustrate that even moderate relaxation from the crystal structure can introduce structural changes that lead to shifts of more than 4 kcal mol^−1^ in calculated energies for the methyl transfer reaction.

### 3.2 | Clustering-Based Models

To further evaluate the impact of initial geometries on QM-cluster calculations, we analyzed the 40 MD-derived structures obtained from hierarchical clustering. The selected frames for QM calculations are highlighted with red dots in Figure 2. Although many of these frames appear early in the trajectory, their distribution across distinct RMSD regions confirms that the clustering approach sampled diverse conformations for subsequent QM calculations.

**FIGURE 3 |:**
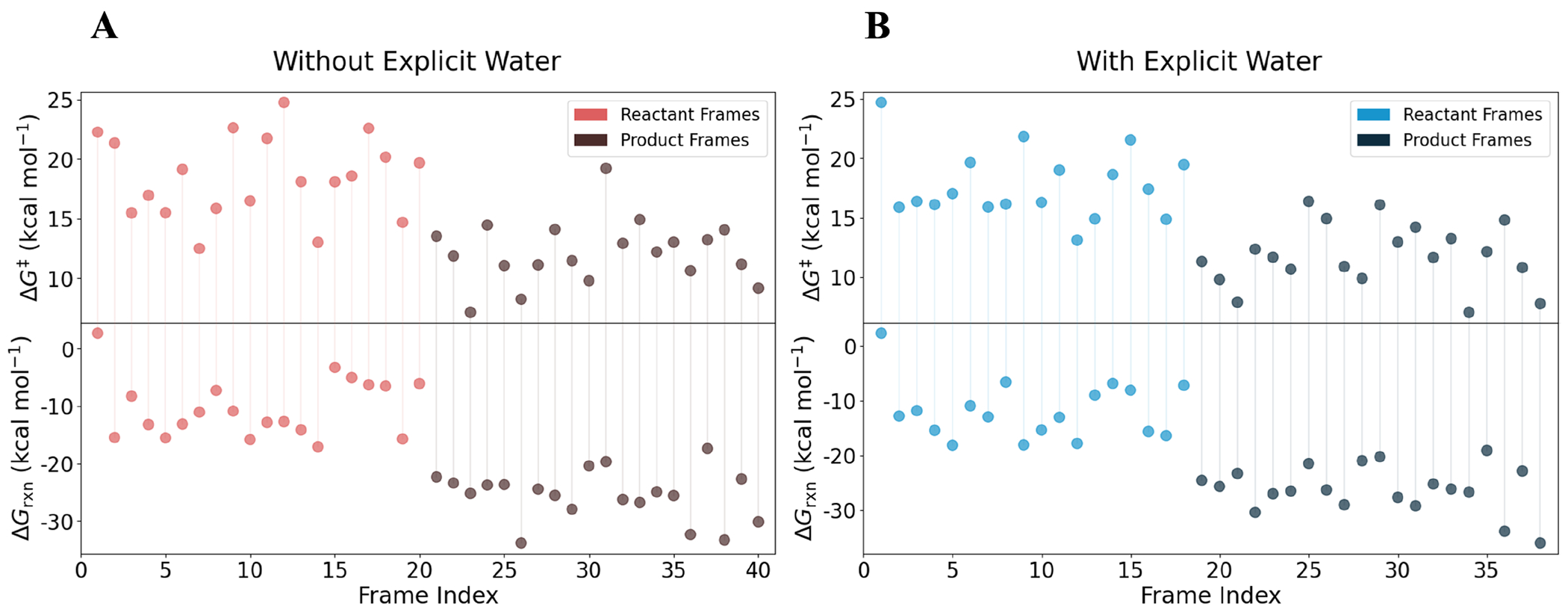
Gibbs free energies of activation (Δ*G*^‡^, top panels) and reaction (Δ*G*_rxn_, bottom panels) computed from large QM-cluster models constructed from 40 reactant- and product-state MD frames. Plot A corresponds to models without explicit solvent, while Plot B includes explicit water molecules in the QM region that interact with SAM or Glycine.

Figure 3 summarizes the computed free energies of activation and reaction for the QM-cluster models generated from both reactant- and product-state MD frames, treated either with or without explicit first-shell solvent molecules. The detailed data can be found in the Supporting Information (Tables S1–S4).

For the models without explicit solvent (Figure 3A), the QM energies of models constructed from the reactant-state frames are shown in red, while those from product-state frames are shown in dark-brown. The upper panel of Figure 3A shows the free energies of activation (Δ*G*^‡^), which ranges from 7.00 to 25.00 kcal mol^−1^, with a mean of 15.32 kcal mol^−1^ and a standard deviation of 4.35 kcal mol^−1^. The lower panel shows the corresponding reaction free energies (Δ*G*_rxn_), which are more widely distributed, from −34.00 to +3.00 kcal mol^−1^ with a mean of −17.93 kcal mol^−1^ and a standard deviation of 8.88 kcal mol^−1^.

For the models with explicit waters (Figure 3B) which were built from the same set of MD frames, the reactant- and product-state QM-cluster model energies are shown in blue and dark-blue. The upper panel likewise shows the Δ*G*^‡^, which also ranges from 7.06 to 24.70 kcal mol^−1^, with a mean of 14.62 kcal mol^−1^ and a standard deviation of 3.99 kcal mol^−1^. The lower panel shows the corresponding Δ*G*_rxn_, which ranges from −36.07 to 2.40 kcal mol^−1^ with a mean of −19.37 kcal mol^−1^ and a standard deviation of 8.54 kcal mol^−1^.

As noted in the Method section, when explicit water molecules were included in the QM calculations, two TSs could not be located. The corresponding models without explicit water have Δ*G*^‡^ of 21.74 and 24.76 kcal mol^−1^; Δ*G*_rxn_ of −12.84 and −12.71 kcal mol^−1^. Their exclusion may have slightly lowered the statistical mean but did not change the overall distribution.

An important aspect of both datasets is the wide spread observed in both free energies of activation and reaction (Table 3). Compared to the four models that closely resemble the X-ray crystal structure, the broader set of MD-derived conformations reveals that the variation is driven by the enzyme’s inherent conformational flexibility. These energetic differences reflect a broader trend consistent with the dynamic nature of biological macromolecules. Some conformations are more thermodynamically favorable than others, and certain conformations are better preorganized for methyl transfer, resulting in lower activation barriers.

These results underscore a fundamental limitation of single-structure approaches: computed energetics can be sensitive to both the choice of QM region and the extent of conformational sampling [20], meaning that no single free energy of activation or reaction should be interpreted as uniquely “correct.” The outcome, to a large degree, is highly dependent on the starting geometry used in the calculation. Although a single conformation can reproduce the correct bond-breaking/bond-forming pattern, quantitative mechanistic interpretations (e.g., how strongly specific residues stabilize the transition state) may not generalize beyond that structure, especially when the QM-model is built from a frame that is an outlier relative to the dominant population sampled for the reaction. The broader distribution observed here underscores the need for extensive conformational sampling to obtain reliable energetic insight.

In addition to the overall spread in the energies, a notable difference is observed between the QM results obtained from reactant-state and product-state starting structures. In the upper panels of Figure 3, the points corresponding to reactant-state frames are generally located higher than those of the product-state frames, indicating that most of the higher activation barriers arise from the reactant ensemble in both with and without explicit-solvent models.

For free energies of activation without explicit waters, the reactant-state frames exhibit a higher average barrier of 18.48 kcal mol^−1^, whereas the product-state frames yield lower values, averaging 12.17 kcal mol^−1^ and typically falling below the overall mean of 15.32 kcal mol^−1^. The results of models with explicit-solvent follow the same trend, with the reactant-state barriers remaining systematically higher than those of the productstate frames. A similar pattern appears in the lower panels for the reaction free energies (Figure 3), where the product-state points lie lower on average. These systematic differences indicate that conformations sampled from product-state simulations are more frequently arranged to stabilize both the transition state and the product, whereas reactant-state frames tend to be less favorable for the overall reaction. Although the QM models are comparable in size and residue composition (see Tables S1–S4 in the Supporting Information for number of residues and frozen atoms information), the energetic trends vary substantially across frames. These differences most likely arise from variations in the spatial arrangement and relative positioning of key residues and atoms within the active site.

### 3.3 | Comparison of QM Calculations With and Without Explicit Water Molecules

Having established that both datasets display a wide range of activation and reaction free energies, we next examined how the inclusion of explicit solvent influences these results.

When compared directly with the results of models without explicit water molecules, the inclusion of first-shell water molecules produces only small shifts in the calculated energies (Table 3). The mean activation free energy decreases slightly from 15.32 to 14.62 kcal mol^−1^, while the mean reaction free energy becomes more exergonic (from −17.93 to −19.37 kcal mol^−1^). Despite these shifts, the overall spread of both quantities remains nearly identical (Figure 3), indicating that explicit solvation does not alter the wide energy variation that arises from conformational diversity in the MD frames.

This observation is likely specific to the GNMT catalyzed reaction. In our QM-cluster models, the included first-shell water molecules do not directly participate in the methyl-transfer event or undergo substantial state specific reorganization. Instead, they provide similar environmental stabilization to the reactant, transition state, or product. As a result, their contributions largely cancel in the computed free energy differences. This interpretation is consistent with the fact that the reaction occurs within a well-defined active-site that is largely shielded by the cofactor and surrounding residues, limiting solvent exposure.

**FIGURE 4 |:**
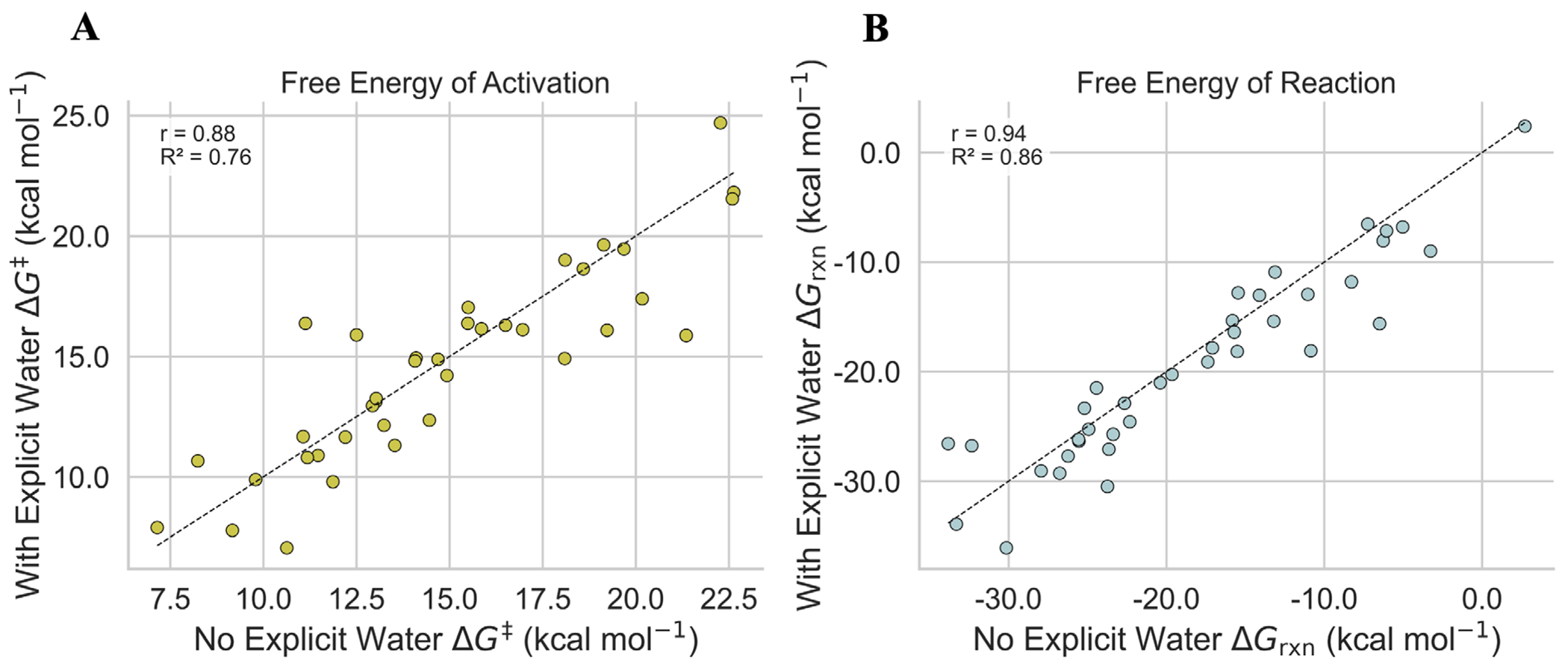
Comparison of activation (Δ*G*^‡^, Plot A) and reaction (Δ*G*_rxn_, Plot B) free energies computed from QM cluster models built without explicit water molecules and with explicit first-shell water molecules. Each point corresponds to a matched MD-derived frame used in both QM calculations. Dashed lines represent 1:1 correspondence between the two treatments.

Building upon the statistical comparison summarized in Table 3 and Figure 3, a direct frame-by-frame correlation analysis was carried out to evaluate how closely the results obtained with and without explicit solvent align (Figure 4).

For the Gibbs free energy of activation (Figure 4A), the points lie close to the 1:1 line (*r* = 0.88, *R*^2^ = 0.76), indicating that frames with higher barriers in the models without explicit waters also yield proportionally higher barriers when explicit water molecules are included. The vertical scatter is generally small, typically within 2–3 kcal mol^−1^, although a few frames show deviations up to 5 kcal mol^−1^. The reaction free energies (Figure 4B) show an even stronger correlation (*r* = 0.94, *R*^2^ = 0.86), which demonstrates that adding explicit first-shell water molecules preserves the relative energetic ordering across frames just as closely as the activation energies. Overall, the strong linear correlations between the two datasets indicate that adding explicit water introduces only small shifts in absolute energies while leaving the frame-to-frame energetic relationships essentially unchanged.

### 3.4 | Potential Energy Surface and Structural Origin of Variation

**FIGURE 5 |:**
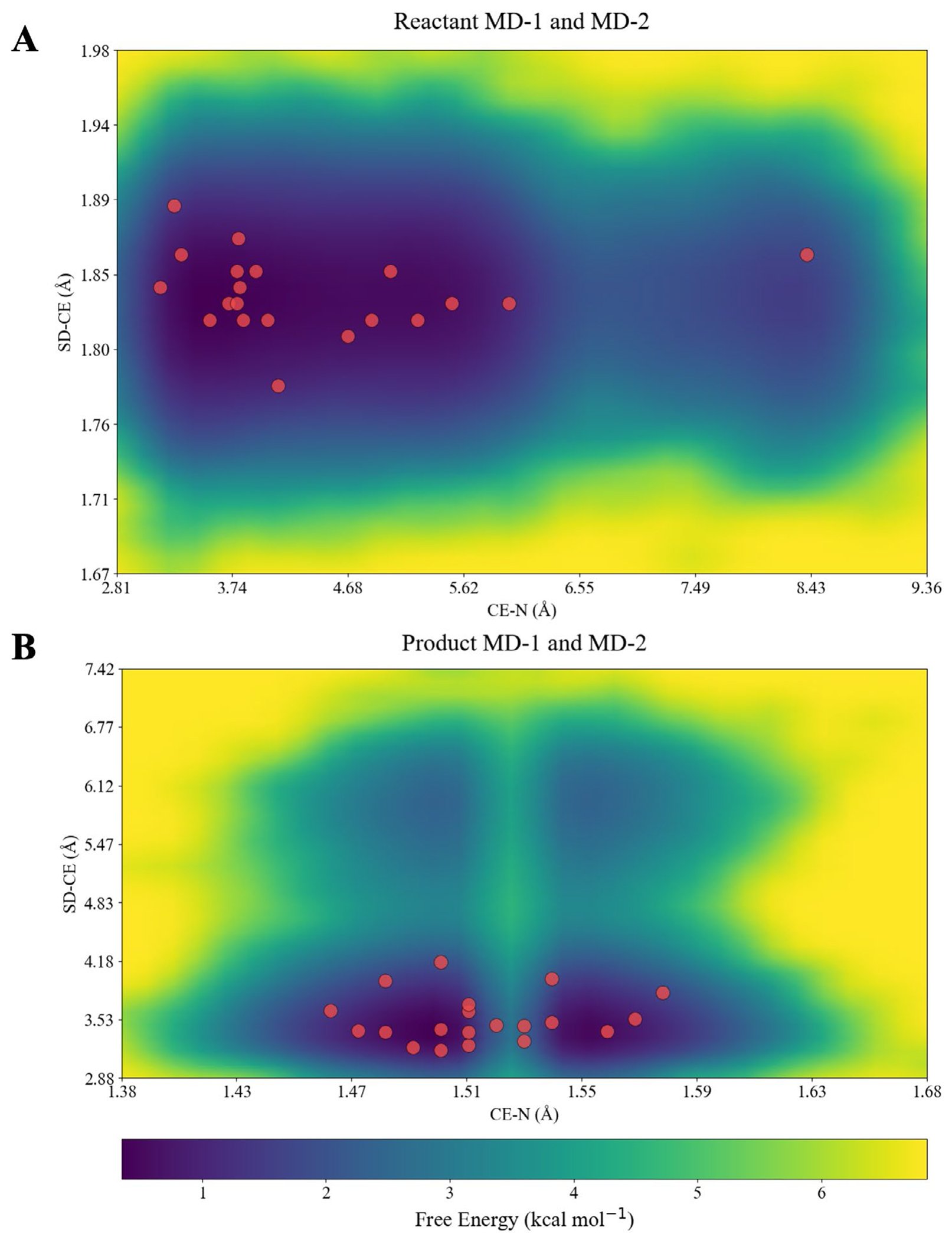
Potential energy surfaces (PES) derived from (A) reactant- and (B) product-state MD simulations of GNMT, plotted along the SD–CE and CE–N reaction coordinates. The color maps represent the configurational free energy landscapes obtained from the MD trajectories using the Boltzmann relation *F* =−*k*_B_ *T* ln(*p*). Overlaid red points correspond to representative QM-cluster models extracted from the same simulations, showing the positions of frames used for subsequent QM calculations.

To determine whether the overall spread in energies could be associated with simple geometric features of the active site, we projected the MD ensembles onto potential energy surfaces (PES) defined by the distance between the SAM sulfur donor and the transferred methyl (SD–CE) and between the methyl and the glycine acceptor nitrogen (CE–N) (Figure 5). The bond distances are shown in Table 4. These two coordinates were chosen because they correspond directly to the bond-breaking and bond-forming events of the methyl transfer mechanism and therefore provide a direct structural basis for interpreting the mechanism.

As shown in Figure 5A and Table 4 (reactant), the reactant-state frames exhibit a broad geometric distribution, with CE–N distances ranging from approximately 3.16 to 8.39 Å, while the SD–CE coordinate remains narrowly constrained between 1.78 and 1.89 Å. This limited spread in SD–CE arises because these structures represent the reactant configuration, in which the sulfur atom of SAM remains covalently bound to the methyl carbon (CE); as a result, the sulfur-carbon linkage is essentially fixed near its equilibrium bond length.

Across the reactant frames (Table 4), the computed free energies of activation (12.50 to 24.76 kcal mol^−1^) and reaction (−17.09 to +2.70 kcal mol^−1^) show no obvious dependence on both coordinate for either QM calculations with explicit water and no explicit water. Among structures with nearly identical CE–N distances around 3.80 Å and similar SD–CE values near 1.83 Å, several frames yield high barriers whereas others are distinctly low. For example, in the no-explicit-water calculations, Frames 2 (3.83, 1.82 Å), 17 (3.80, 1.84 Å), and 18 (3.78, 1.85 Å) give activation free energies of 21.35, 22.59, and 20.17 kcal mol^−1^, respectively, while Frame 7 (3.79, 1.87 Å) has a much lower value at 12.50 kcal mol^−1^, and Frame 14 (3.56, 1.82 Å) remains comparable at 13.02 kcal mol^−1^. The activation free energies for these geometrically similar frames thus differ by nearly 10 kcal mol^−1^.

In contrast, the product-state structures form a far more compact cluster in the CE–N coordinate (Figure 5B; Table 4, product), with values between 1.46 and 1.58 Å, while the SD–CE spans a broader range of about 3.2–4.2 Å. The larger spread in the SD–CE coordinate for the product frames compared to the reactant arises naturally from the transfer of the methyl group from sulfur to nitrogen; once methylation is complete, the sulfur-carbon bond is broken and the sulfur atom moves further from the methyl carbon. Frames 8 (1.52, 3.47 Å), 11 (1.53, 3.46 Å), and 13 (1.46, 3.63 Å) lie within a narrow region of the CE–N and SD–CE coordinates, yet give some of the higher activation free energies in the product ensemble. In contrast, Frames 6 (1.51, 3.62 Å), 10 (1.51, 3.70 Å), and 20 (1.54, 3.50 Å) have similar geometric features (Figure 5) but yield significantly (8 to 10 kcal mol^−1^) lower barriers.

For the reaction free energies, frames with similar CE–N and SD–CE values generally give similar Δ*G*_rxn_, even though the overall range across the ensemble is larger than for the activation barriers. Notable exceptions are product Frames 8 (1.52, 3.47 Å) and 11 (1.53, 3.46 Å), which have almost identical geometries yet differ in their reaction free energies by about 6 kcal mol^−1^ for both solvation treatments.

This lack of correlation indicates that the SD–CE and CE–N distances of the starting MD frames alone are insufficient to describe the variations in downstream QM energies, even though these coordinates provide direct mechanistic interpretation of the bond-breaking and bond-forming events. The determinants of barrier heights and reaction free energies instead arise from more complex combinations of subtle geometric and interaction-based features that shape how a given MD conformation influences the outcome of the calculations. A similar conclusion was reported by Świderek et al. [58], who showed that variations in the methyl donor–acceptor distance in GNMT and its Tyr21 mutants are small and do not correlate with the observed changes in activation free energies or kinetic isotope effects. To explore whether broader conformational variations of the enzyme distinguish the reactant and product ensembles and contribute to the observed spread in QM energies, we next performed PCA on the MD trajectories.

### 3.5 | Principal Component Analysis of Conformational Space

PCA [55, 56] was performed on the protein C_α_ atoms after aligning each frame to the reference X-ray crystal structure. The MD trajectories from the reactant- and product-state simulations, which were also used in the 2D PES analysis, were concatenated into a single combined ensemble before constructing the covariance matrix. The analysis was restricted to protein C_α_ atoms, with the SAM cofactor and glycine substrate excluded, so that the resulting principal components reflect the dominant collective backbone motions sampled during the MD simulations.

**FIGURE 6 |:**
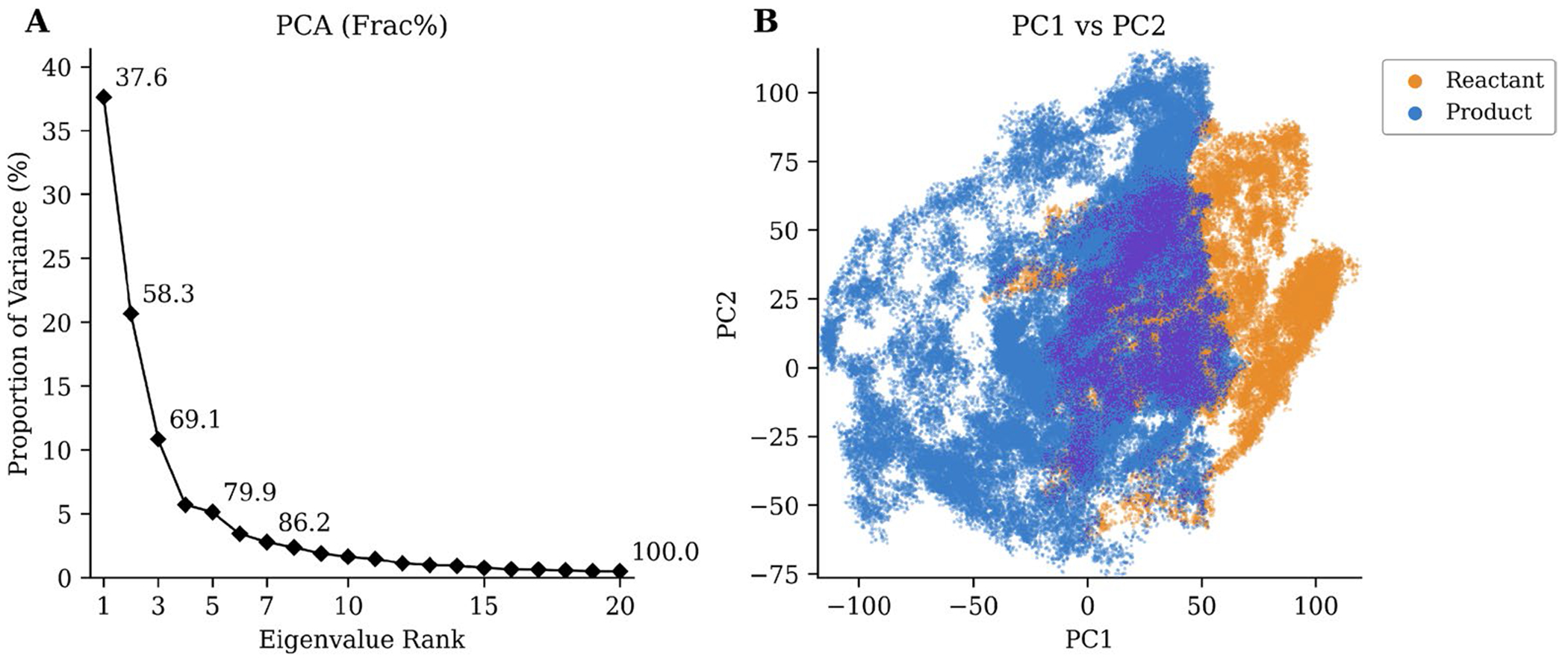
Principal component analysis of the combined MD trajectories. (A) Scree plot showing the fraction of C_α_ positional variance captured by the first 20 principal components (PC1 = 37.6%, PC2 = 20.7%; cumulative PC1–PC2 = 58.3%). (B) Projection of all MD frames onto the PC1–PC2 subspace. Reactant- (orange) and product-state (blue) simulations occupy distinct regions of conformational space, with separation occurring primarily along PC1. The purple region indicates areas of conformational overlap between both states.

As shown in Figure 6A, PC1 accounts for 37.6% of the total C_α_ positional variance of the protein backbone, while PC2 accounts for an additional 20.7%, such that the first two components together capture 58.3% of the overall conformational variance represented by the protein C_α_ coordinates. Projection of all MD frames onto the PC1–PC2 subspace (Figure 6B) reveals that reactant- and product-state simulations populate distinct, though partially overlapping, regions of backbone conformational space. The primary distinction occurs along PC1: reactant simulations cluster toward higher PC1 values, whereas product simulations extend toward lower PC1 values. In contrast, PC2 contributes to the overall spread of conformational sampling but does not differentiate the two states to the same extent. These observations indicate that the dominant collective backbone motions described by PC1 capture the main structural difference between the reactant and product ensembles.

**FIGURE 7 |:**
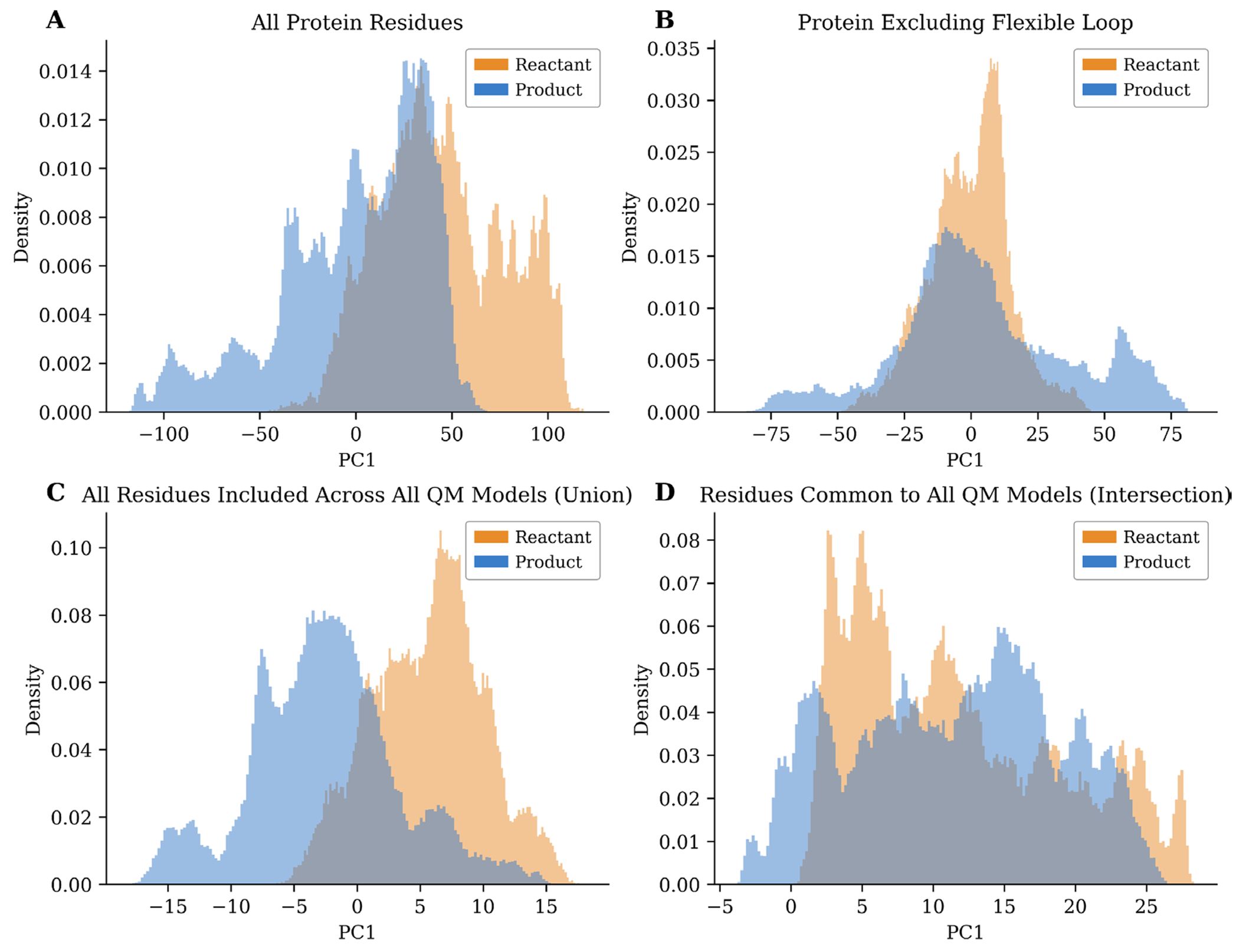
Distribution of the first principal component (PC1) for reactant- (orange) and product-state (blue) MD trajectories under different structural representations. (A) PCA performed on C_α_ atoms of all protein residues. (B) PCA performed on the protein excluding residues in the flexible loop region. (C) PCA restricted to the union of all residues included across the QM-cluster models. (D) PCA restricted to the intersection of residues common to all QM models.

To further examine how the dominant conformational coordinate depends on the structural region included in the analysis, PCA was performed using four different residue selections, and the resulting PC1 distributions for reactant- and product-state frames were compared (Figure 7). When PCA was performed using the C_α_ atoms of all protein residues (Figure 7A), the PC1 distributions of the reactant and product ensembles show moderate overlap, particularly in the range of approximately −25 to 70 along PC1. However, the two ensembles display distinct population densities along this coordinate, with reactant frames populating higher PC1 values and product frames sampling lower PC1 values with a broader distribution extending toward negative PC1. These differences in both population density and sampling range are consistent with the separation observed in the PC1–PC2 projection. Excluding residues in the flexible loop region (Figure 7B) reduces this population shift. Under this representation, the reactant and product distributions become centered near similar PC1 values with strongly overlapping density profiles, indicating that motions of the flexible loop contribute substantially to the state-dependent conformational differences captured by the full-protein PCA. Focusing on the active-site region, PCA was next performed using the union of residues included across all QM-cluster models (Figure 7C). The overall spread of PC1 values is substantially reduced, with most frames confined to a narrower range of approximately −20 to 20 along PC1. The reactant and product distributions again show a modest shift along PC1, although the density peaks remain closer together and the overlap between the ensembles is greater than that observed for the full-protein representation. Finally, when PCA was restricted to the intersection of residues present in every QM-cluster model (Figure 7D), the PC1 range is further constrained, approximately −4 to 30, and the distributions become broad with extensive overlap and similar density profiles for both ensembles, indicating that little distinct separation remains under this most restrictive active-site definition.

**FIGURE 8 |:**
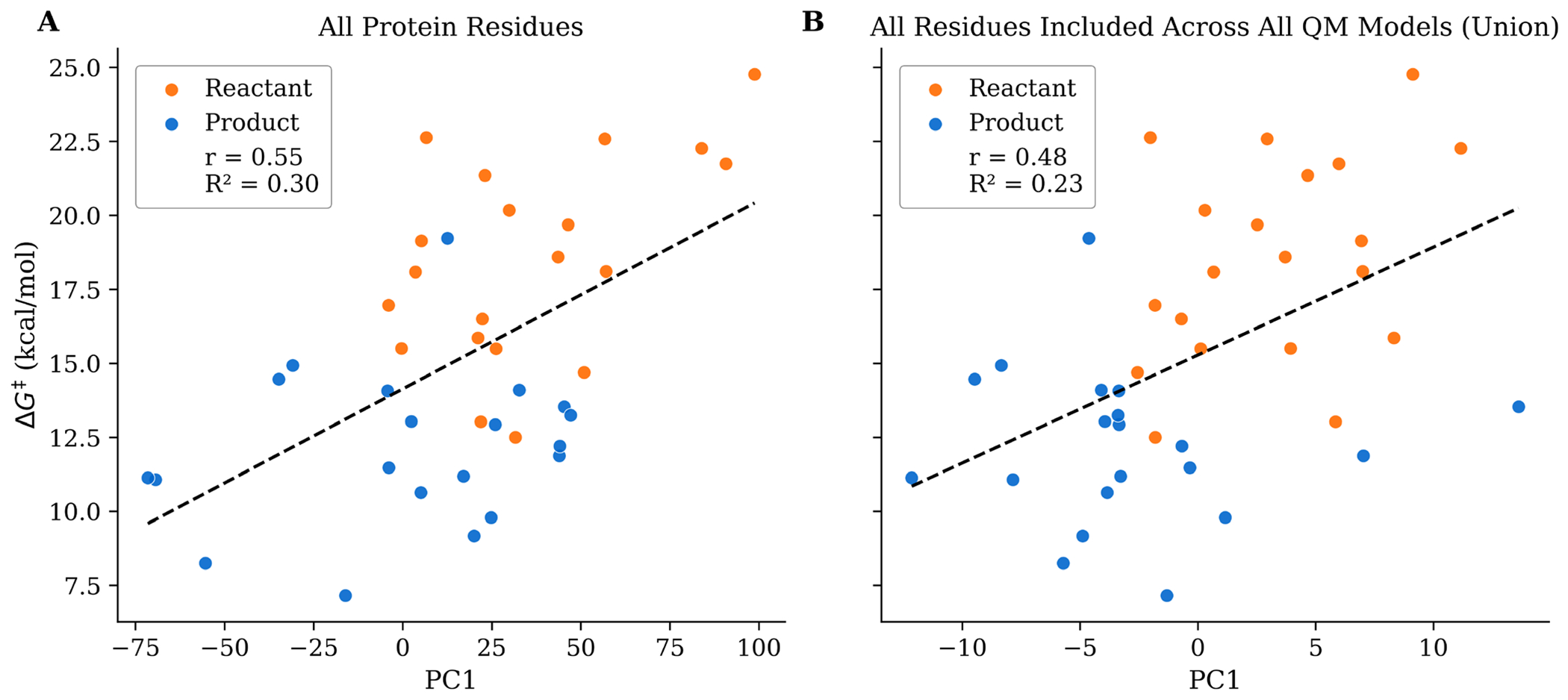
Correlation between PC1 values and QM-computed activation free energies (Δ*G*^‡^) for QM-cluster geometries derived from reactant-initiated (orange) and product-initiated (blue) MD simulations. (A) PC1 obtained from PCA performed on all protein C_α_ atoms. (B) PC1 obtained from PCA restricted to the union of active-site residues included across all QM-cluster models. Dashed lines represent linear least-squares fits. Pearson correlation coefficients (*r*) and corresponding coefficients of determination (*R*^2^) are shown within each panel.

Based on the PC1 distributions of reactant- and product-state simulations across these four residue selections, the conformational differences between the two ensembles become much less pronounced when the analysis is restricted to active-site residues. This suggests that the dominant PC1 signal is primarily driven by broader protein motions and is therefore less effective at resolving subtle conformational differences within the active-site that may contribute to the variation in computed QM energies. As shown in Figure 8, the PC1 values of the QM frames show a moderate positive correlation with the computed activation free energies (Δ*G*^‡^) when PCA is performed using all protein C_α_ atoms (*r* = 0.55, *R*^2^ = 0.30) and when PCA is restricted to the active-site union residue set (*r* = 0.48, *R*^2^ = 0.23).

To examine whether including additional backbone and side-chain atoms alters the PCA description of the active-site region, PCA was repeated for the active-site union and intersection residue sets using all heavy atoms rather than only C_α_ atoms. The resulting PC1 distributions and PC1-Δ*G*^‡^ correlations were qualitatively similar to those obtained from the C_α_-based analysis (see Supporting Information, Figures S2 and S3), indicating that the dominant conformational motions are already captured by the C_α_ representation.

Although PC1 shows a moderate correlation with the computed activation barriers, frames with similar PC1 values can still yield substantially different QM activation energies. This suggests that PC1 alone is insufficient to explain the energetic variation, motivating the use of more informative structural descriptors and data-driven approaches.

### 3.6 | Machine Learning Performance Using Donor–Methyl–Acceptor Distances

Using two datasets, one without explicit water molecules and one with explicit water molecules, we first evaluated how well the fundamental geometric descriptors of the methyl transfer reaction, namely the SD–N, CE–N, and SD–CE distances extracted from the MD frames can be used to predict the activation and reaction free energies. The randomly selected 35 frames are used as the testing dataset for this evaluation. Together, these three distances provide a more complete geometric description of the donor–methyl–acceptor arrangement. Because machine learning models can readily accommodate multidimensional inputs, this three-coordinate representation offers a straightforward starting point for regression analysis.

The machine learning results in Table 5 show that the models trained on the three key geometric descriptors of the methyl transfer reaction exhibit comparable predictive performance across datasets with and without explicit water molecules. However, the resulting structure–energy relationships differ clearly between models that include explicit waters and those that do not.

When explicit water molecules are not included, the best-performing models for predicting the activation free energy are the linear regression based models. Elastic Net, Lasso, Ridge, and Linear Regression all yield MAEs between 2.67 and 2.85 kcal mol^−1^, with Dummy-relative improvements between 12% and 18%. In contrast, the nonlinear models exhibit weaker performance using the same dataset. Random Forest, AdaBoost, Gradient Boosting, and Decision Tree all exhibit relatively high MAEs ranging from 3.24 to 3.96 kcal mol^−1^. Correspondingly, these models show negative improvements relative to the Dummy baseline with values decreasing from 0% to approximately −22%.

These negative percentage improvement values indicate that they perform worse than a baseline predictor that simply returns the mean of the target values and therefore fail to capture meaningful geometric–energy relationships. Nonlinear model KNN performs slightly better, with a modest positive improvement of 10%, but it still does not outperform the regularized linear models (Ridge, Lasso and Elastic Net), which constrain coefficient magnitudes to reduce overfitting. These results suggest that, when no explicit water molecules are present, the distance descriptors primarily follow a largely linear global relationship with Δ*G*^‡^.

In the models that include explicit water molecules, the performance trends across algorithms are significantly different. For Δ*G*^‡^, every model shows a positive improvement relative to the Dummy baseline. The linear models remain strong performers, with Elastic Net, Lasso, and Ridge yielding MAEs between 2.60 and 2.79 kcal mol^−1^. KNN now achieves the lowest MAE at 2.57 kcal mol^−1^, corresponding to a 24.3% improvement, and the tree-based methods, which performed below baseline when no explicit water molecules were included, now show improvements between 14% and 19%. Although the MAEs for the datasets without and with explicit water molecules are comparable, the relative performance compared to the Dummy baseline is substantially and consistently higher when explicit water molecules are included. This indicates that explicit water molecules enhance the geometric signal contained in the data and introduce additional structural information that machine learning models can more effectively exploit.

For Δ*G_rxn_*, both datasets show that all machine learning models outperform their respective Dummy baselines (6.21 kcal mol^−1^ for no explicit water and 7.12 kcal mol^−1^ for explicit water). When no explicit water molecules are included, improvements range from 0.91% to 30.65%, with MAEs spanning 4.31–6.15 kcal mol^−1^.

Models trained on the dataset that includes explicit water molecules perform systematically better than their counterparts trained without explicit waters, with the exception of AdaBoost, which does not follow the trend. In the explicit water case, KNN shows the strongest performance, achieving an MAE of 3.88 kcal mol^−1^ and a 45.48% improvement compared to the Dummy baseline, while all other models exhibit improvements between 29.43% and 41.12%, with MAEs spanning 4.19–5.03 kcal mol^−1^. Although adding explicit water molecules increases the overall spread of the reaction free energies, as indicated by higher Dummy baseline errors (6.21 kcal mol^−1^ without explicit water and 7.12 kcal mol^−1^ with explicit water), it strengthens the correlation between the donor-methyl-acceptor distances and the reaction energies.

These numerical trends can be rationalized by the inclusion of explicit water molecules that interact directly with SAM and the glycine in the active site, modulating both the local structural and reaction environment. Importantly, the SD-CE, CE-N, and SD-N distances used as descriptors are extracted directly from MD frames and do not correspond to bond lengths optimized in QM reactant, transition state, or product structures. Instead, they describe the instantaneous geometries sampled along the trajectory, which serve as input for the subsequent QM energy computations. Variations in the MD-derived distances capture differences in bond stretching, donor-acceptor alignment, and overall active site compression, all of which influence the computed QM cluster energies.

When no explicit water molecules are included, solvent effects are spatially averaged, smoothing out local fluctuations and yielding a more global, approximately linear relationship between geometry and energy that linear and regularized models capture well. In contrast, when explicit water molecules are present, specific hydrogen bonding and water-mediated polarization introduce additional, conformational-dependent energy contributions. These effects are reflected in the enhanced performance of nonlinear and local learners such as KNN and ensemble tree-based models. The observed shift of tree-based methods from negligible or negative improvement in no explicit water dataset to clear positive improvement in the explicit water dataset supports the conclusion that the geometry energy relationship becomes more structured and more strongly correlated to the local environment when explicit waters are present.

In addition to MAE, the coefficient of determination (*R*^2^) was also evaluated, and the corresponding results are listed in Table S5. In this dataset, the *R*^2^ values are consistent with the trends from the %MAE improvement metrics, with better-performing models generally yielding higher *R*^2^ values.

### 3.7 | Combined Analysis of the Donor–Methyl–Acceptor Distance and Solvent-Derived Feature Representations

Having established that the geometric descriptors of the methyl transfer reaction (SD–CE, CE–N, and SD–N distances) exhibit predictive performance in the datasets, we next examined whether additional structural information could better capture the relationship between geometry and energy, particularly in the dataset containing explicit water molecules. Two feature representations were therefore evaluated: the combined distance–solvent representation, which merges the donor–methyl–acceptor distance representation with the solvent-derived representation described in the Computational Methods section, and the solvent-derived representation alone. These analyses allow us to assess the extent to which solvent structure, independent of the methyl-transfer coordinate, contributes to the predictability of the explicit water model energies.

The combined distance–solvent representation produces only limited improvement in predictive accuracy for both Δ*G*^‡^ and Δ*G*_rxn_ (Table 6). For activation energies, the best performing model (AdaBoost) achieves an MAE of 2.40 kcal mol^−1^, representing a modest improvement over the best model trained solely on the donor–methyl–acceptor representation reported earlier (2.57 kcal mol^−1^ from KNN trained on the explicit water dataset; Table 5). This corresponds to a relative improvement of 29.1% (Table 6), only slightly higher than the 24.3% obtained using the distance representation alone. The remaining models trained on the combined distance–solvent features have a narrow MAE range of 2.55–2.76 kcal mol^−1^ which is only a small shift compared to the 2.57–2.89 kcal mol^−1^ range observed for the distance representation (Table 5). Together, these comparisons indicate that the inclusion of the solvent-derived representation does not substantially enhance the overall predictive accuracy beyond what is achieved by the methyl-transfer distances alone.

For reaction free energies, models trained on the combined distance–solvent representation yield results that are nearly identical to those obtained using the donor–methyl–acceptor distances alone. KNN again provides the lowest MAE at 3.88 kcal mol^−1^, corresponding to a 45.48% improvement relative to the Dummy baseline, which matches the performance observed previously when only the three methyl transfer distances were used (Table 5). Nearly all other models trained on the combined distance–solvent representation fall within an MAE range of 4.26–6.20 kcal mol^−1^ which closely mirrors the 4.19–5.03 kcal mol^−1^ range obtained from the distance representation. These results indicate that incorporating solvent-derived descriptors does not provide an additional predictive advantage for Δ*G*_rxn_. Instead, the information contributed by solvent configuration appears to largely overlap with what is already encoded by the SD–N, SD–CE, and CE–N geometric features.

### 3.8 | Analysis of the Solvent-Derived Feature Representation

In contrast, models trained on only the solvent-derived representation show a clear reduction in predictive performance for both energy targets. For Δ*G*^‡^, Ridge regression achieves the lowest MAE of 2.78 kcal mol^−1^ (Table 6), corresponding to a 17.92% improvement relative to the Dummy baseline. This represents a decrease of approximately 6%–11% compared to the 23%–29% improvements achieved by the best models trained on the combined distance–solvent representation. Furthermore, most models trained on solvent–derived representation perform close to the Dummy baseline, with Decision Tree showing only a 0.06% improvement and several others remaining below 10%, indicating that solvent representation alone does not encode sufficient structural information to reliably predict the free energies.

For Δ*G*_rxn_, models trained on the solvent-derived representation exhibits a somewhat stronger predictive signal compared to their performance on Δ*G*^‡^, but they still underperform relative to models trained on the donor–methyl–acceptor representation. Within the solvent-derived feature set, KNN performs best, achieving an MAE of 4.50 kcal mol^−1^ and a 36.88% improvement relative to the Dummy baseline. Random Forest and Decision Tree follow closely, both showing improvements above 30%. These results indicate that aspects of the solvent configuration do correlate with the reaction free energies; however, this performance remains well below that achieved using either the combined distance–solvent representation or the methyl transfer distance features, where improvements of 40%–46% are routinely observed. Notably, Linear and Ridge regression perform below the Dummy baseline (−22.53% and −9.11%, respectively), while other regularized linear methods show moderate improvements (19%–26%), indicating that the solvent-derived features contain limited linear signal that is insufficient to capture most of the variation in the energies.

Taken together, these results demonstrate that the local solvent environment contains only limited predictive information for both Δ*G*^‡^ and Δ*G*_rxn_. Adding solvent-derived descriptors to the methyl transfer distance features produces only marginal improvements, while excluding the methyl transfer based geometry results in a systematic decrease in accuracy across all algorithms. The solvent-derived representations contain a limited predictive signal, which is better captured by nonlinear models, as seen from the KNN and tree-based model results for Δ*G*_rxn_. By contrast, the consistently higher accuracy obtained from models that include the donor–methyl–acceptor distances confirms that these features remain the dominant structural predictors of both activation and reaction free energies in this system. Similar overall trends are also observed in the corresponding *R*^2^ values (Table S6).

### 3.9 | Alternative Structural Representations

In this section, we focus on the dataset without explicit water molecules to enable a clearer and more controlled evaluation of the alternative structural representations. In the explicit-water dataset, the number, identity, and spatial arrangement of retained water molecules vary substantially across frames, making their structural contributions highly heterogeneous and difficult to compare consistently between models. This variability complicates any attempt to isolate the effect of specific feature representations. By contrast, the no-explicit-water dataset provides a more uniform structural baseline in which the dominant environmental contributions arise from the enzyme and active-site residues themselves. Focusing on this condition therefore allows us to more directly assess whether broader residue-based geometry and interaction-pattern features can capture meaningful enzyme-driven contributions to the energies, independent of fluctuating solvent structure.

The results in Table 7 show that expanding the descriptor set beyond the donor–methyl–acceptor coordinates provides limited benefit for predictive performance. When trained on the pairwise-distance features, several models achieve moderate accuracy gains, with Δ*G*^‡^ MAEs clustering around 2.40–2.80 kcal mol^−1^ and Δ*G*_rxn_ MAEs typically in the 4.10–5.10 kcal mol^−1^ range. Although these values represent improvements over the Dummy baseline, they are broadly comparable to those trained with the simpler donor-based descriptors without water, where the best performing models achieve Δ*G*^‡^ MAEs ranging from 2.60 to 2.80 kcal mol^−1^ and Δ*G*_rxn_ MAEs of approximately 4.30–4.60 kcal mol^−1^ (Table 5).

Similarly, for the interaction-type representation, most models achieve Δ*G*^‡^ values between 2.70 and 2.90 kcal mol^−1^, and Δ*G*_rxn_ values near 4.20–4.60 kcal mol^−1^, corresponding to improvements of approximately 10%–33% over the Dummy baseline. And similar overall trends are observed in the corresponding *R*^2^ values (Table S7). While this indicates that categorical interaction patterns encode a measurable predictive signal, the model accuracy obtained from this feature set does not exceed that achieved using either the pairwise-distance features or the donor–methyl–acceptor distance descriptors.

Overall, both alternative representations yield predictive accuracies that are comparable to but not significantly better than those obtained using the minimal methyl-transfer features. Despite their higher dimensionality, these feature sets do not provide consistent improvements in performance, and in some cases introduce reduced performance across algorithms. Given the size of the dataset, the low-dimensional SD–N, SD–CE, and CE–N distance features represent a more efficient and robust choice for modeling both the free energies of activation and reaction. However, the machine-learning models employed in this study remain limited by the simplicity of these descriptors, which restricts their ability to present broader aspects of the enzyme’s conformational environment. Furthermore, because these features were specifically designed based on the chemistry of this system, they may not generalize readily to other enzymes or reaction types. These limitations highlight the potential need for more flexible machine-learning architectures that can more easily adapt across systems, incorporate higher-order structural information, and learn latent relationships directly from the data. Such approaches may ultimately enable more comprehensive analysis of structure–energy relationships, providing insights that are both broadly applicable and mechanistically informative.

## 4 | Conclusion

In this work, we systematically examined how variations in starting geometry influence the computed energetics of the GNMT catalyzed methyl-transfer reaction, while the underlying mechanism remained a single-step process.

We began by assessing how brief MD relaxations of the GNMT crystal structure affect the computed reaction energetics, relative to previously reported QM results obtained directly from the unrelaxed conformation. Despite using essentially the same computational setup and model-building procedure, the early stage relaxed structures which deviated by approximately 2.3–2.8 Å RMSD from the structure used in the earlier study produced significantly higher activation free energies (by 4.7–7.4 kcal mol^−1^) and reaction free energies (by 0.0–5.7 kcal mol^−1^). These shifts highlight the sensitivity of downstream QM results to the starting geometry and motivated a broader investigation into how a diverse ensemble of starting structures may impact the calculated energies.

Building on this initial observation, we expanded our analysis to conformational ensembles obtained from the four (two initiated from the reactant state and two initiated from the product state) independent MD trajectories, using frames selected through clustering and random selection. QM cluster models were subsequently constructed from these frames using RINRUS, with and without first-shell explicit water molecules. Across this broad model set, we observed a wide energetic spread, with Δ*G*^‡^ values spanning roughly 7.0–25.0 kcal mol^−1^ and Δ*G*_rxn_ values ranging from about −36.1 to 3.0 kcal mol^−1^, even though the models were comparable in size and composition. Notably, product-state conformations (with relative shorter SD-N bond distances) consistently yielded lower activation barriers and more favorable reaction energies than those from the reactant-state, reinforcing the importance of geometric preorganization. Furthermore, the inclusion of first-shell explicit water molecules in the QM models had minimal impact on the overall energy distributions, suggesting that the specific stabilizing interactions provided by these waters can be complemented or substituted by alternative residue arrangements in the active site. Consequently, the dominant source of energetic variability arises from differences in the initial geometries rather than from the treatment of local solvation for this enzyme catalytic reaction.

Overall, this work highlights that QM-cluster models built from a single crystal-like conformation cannot capture the intrinsic dynamism of the protein environment, leading to energies that fail to generalize across conformational space. Our findings underscore the importance of integrating conformational sampling and geometric analysis to better reflect the dynamic nature of enzyme reactivity and provide a more reliable basis for mechanistic analysis.

To further probe the determinants of the wide energetic variability, we applied machine-learning models trained on several feature sets, including simple distance descriptors, solvation descriptors, pair-wise distance descriptors, and interaction type descriptors derived on the initial MD frame and the sequential QM-cluster models, and evaluated multiple linear and nonlinear algorithms. Across these experiments, KNN consistently gives the strongest performance, achieving the lowest errors in several feature sets and never performing poorly (above the dummy baseline), while all other models yield MAE ranges of roughly 2.5–4.0 kcal mol^−1^ for Δ*G*^‡^ and 4.0–6.2 kcal mol^−1^ for Δ*G*_rxn_. Our machine-learning results demonstrate that simple distance-based descriptors of the donor–methyl–acceptor arrangement can explain much of the variation in activation and reaction free energies, whereas solvent-derived or higher-dimensional structural features contribute comparatively little. At the same time, our current feature selection protocol involves multiple screening steps to reduce the very large number of initial descriptors and requires several manual decisions, which limits its ability to uncover more complex structure–energy relationships. Future efforts may benefit from employing more expressive molecular representations and flexible machine-learning architectures, which can automatically incorporate a broad range of structural information and offer a richer, more generalizable framework for structure to property relationships in enzymatic reactions.

## Supplementary Material

SI-1

SI-2

SI-4

SI-3

Additional supporting information can be found online in the Supporting Information section. Figure S1: RMSD analysis including the first 16 residues. Figure S2: PC1 distributions based on active-site heavy-atom representations. Figure S3: Correlation between PC1 and computed activation free energies for active-site heavy-atom representation. Table S1: Residues and frozen atoms for QM models derived from reactant-1 frames. Table S2: Residues and frozen atoms for QM models derived from reactant-2 frames. Table S3: Residues and frozen atoms for QM models derived from product-1 frames. Table S4: Residues and frozen atoms for QM models derived from product-2 frames. Table S5: Model performance (*R*^2^) for donor–methyl–acceptor distance features. Table S6: Model performance (*R*^2^) for combined distance–solvent and solvent features. Table S7: Model performance (*R*^2^) for pairwise and interaction-type features. Data S1: jcc70434-sup-0002-Data.zip.

## Figures and Tables

**TABLE 1 |:** Residue composition and frozen atom information for the first set of QM-Cluster models generated by RINRUS.

Residue	Cheng, DeYonker	Model A	Model B	Model C	Model D
Ile17	—	—	—	C_α_	—
Pro18	—	—	—	C_α_	—
Asp19	—	—	—	C_α_	—
Tyr21	C_α_ C_β_	C_α_ C_β_	C_α_ C_β_	C_α_ C_β_	C_α_ C_β_
Trp30	C_α_ C_β_	C_α_ C_β_	C_α_ C_β_	C_α_ C_β_	C_α_ C_β_
Tyr33	C_α_ C_β_	C_α_ C_β_	C_α_ C_β_	C_α_ C_β_	C_α_ C_β_
Ile34	C_α_ C_β_	—	—	—	C_α_ C_β_
Arg40	C_α_ C_β_	C_α_ C_β_	C_α_ C_β_	C_α_ C_β_	C_α_ C_β_
Ala64	C _α_	C _α_	C _α_	C _α_	C _α_
Cys65	C_α_	C_α_	C_α_	C_α_	C_α_
Gly66	C_α_	C_α_	C_α_	C_α_	C_α_
Thr67	—	C_α_	—	—	—
Val69	C_α_ C_β_	C_α_ C_β_	C_α_ C_β_	—	—
Asp70	—	C_α_ C_β_	C_α_ C_β_	C_α_ C_β_	C_α_ C_β_
Asp85	C_α_	C_α_	C_α_	C_α_ C_β_	C_α_
Ala86	C_α_	C_α_	C_α_	C_α_	C_α_
Ser87	C_α_	C_α_ C_β_	C_α_ C_β_	C_α_ C_β_	C_α_
Met90	C _α_	C_α_ C_β_	C_α_ C_β_	C_α_ C_β_	C_α_ C_β_
Ala115	C_α_	C_α_	C_α_	—	C_α_
Asn116	C_α_	C_α_	C_α_	C_α_	C_α_
Trp117	C _α_	C_α_ C_β_	C_α_ C_β_	C_α_ C_β_	C_α_ C_β_
Leu118	—	—	—	C_α_ C_β_	—
Leu136	C_α_	C_α_	C_α_	C_α_	C_α_
Gly137	C_α_	C_α_	C_α_	C_α_	C_α_
Asn138	C_α_	C_α_ C_β_	C_α_ C_β_	C _α_	C_α_
Ser139	C_α_	C_α_ C_β_	C_α_	C_α_	C_α_
His142	C_α_ C_β_	C_α_ C_β_	C_α_ C_β_	C_α_ C_β_	C_α_ C_β_
Leu143	C_α_	C_α_ C_β_	—	—	—
Arg175	C_α_ C_β_	C_α_ C_β_	C_α_ C_β_	C_α_ C_β_	C_α_ C_β_
Tyr194	C_α_ C_β_	C_α_ C_β_	C_α_ C_β_	C_α_ C_β_	C_α_ C_β_
Tyr220	C_α_ C_β_	C_α_ C_β_	C_α_ C_β_	C_α_ C_β_	C_α_ C_β_
Tyr242	C_α_ C_β_	C_α_ C_β_	C_α_ C_β_	C_α_ C_β_	C_α_ C_β_
Tyr283	—	C_α_ C_β_	—	—	—
Number of atoms	432	444	435	424	386
RMSD (Å)	0.000	2.820	2.502	2.693	2.335
Δ*G*^‡^ (kcal/mol)	9.1	16.50	13.84	14.88	15.17
Δ*G*_rxn_ (kcal/mol)	−29.70	−29.73	−21.33	−15.81	−24.02

*Note:* All residue C_α_ atoms and selected residue C_β_ atoms were kept frozen during QM optimization. If a listed residue does not have a specific frozen atom indicated, this means that the residue was not identified by RINRUS as an interacting residue, but its mainchain atoms were included in the QM model and its C_α_ atom was kept frozen following the convention used in the study by Cheng and DeYonker. Each model includes all residues identified as interacting with SAM and glycine. For comparison, the maximal model of Cheng and DeYonker is listed.

**TABLE 2 |:** Summary of data points used for machine-learning analysis.

Model type	Training data points	Test data points	Total
Without explicit water	40	35	75
With explicit water	38	35	73
Total	78	70	148

*Note:* Counts reflect the number of MD frames used in training and testing selected from hierarchical agglomerative clustering and random sampling, respectively.

**TABLE 3 |:** Comparison of activation (Δ*G*^‡^) and reaction (Δ*G*_rxn_) free energies of QM cluster models constructed using the cluster-based MD frames and treated with no explicit solvent (No Exp.) and with first-shell explicit water molecules (Exp.).

	Δ*G*^‡^ (kcal mol^−1^)			Δ*G*_rxn_ (kcal mol^−1^)		
QM model set	Range	Mean	SD	Range	Mean	SD
No Exp. (*n* = 40)	7.00–25.00	15.32	4.35	−34.00–3.00	−17.93	8.88
Exp. (*n* = 38)	7.06–24.70	14.62	3.99	−36.07–2.40	−19.37	8.54

*Note:* Values represent the range, mean, and standard deviation (SD) of all computed data points.

**TABLE 4 |:** CE–N and SD–CE distances (in Å) for selected MD frames, together with the corresponding QM-cluster activation (Δ*G*^‡^) and reaction (Δ*G*_rxn_) free energies (kcal mol^−1^).

	Reactant						Product					
			No explicit water		With explicit waters				No explicit water		With explicit waters	
ID	CE–N	SD–CE	Δ*G*^‡^	Δ*G*_rxn_	Δ*G*^‡^	Δ*G*_rxn_	CE–N	SD–CE	Δ*G*^‡^	Δ*G*_rxn_	Δ*G*^‡^	Δ*G*_rxn_
1	5.24	1.82	22.27	2.70	24.70	2.40	1.58	3.83	13.53	−22.33	11.32	−24.57
2	3.83	1.82	21.35	−15.46	15.88	−12.79	1.56	3.40	11.87	−23.39	9.80	−25.70
3	4.03	1.82	15.49	−8.28	16.38	−11.80	1.50	4.17	7.15	−25.21	7.90	−23.32
4	3.27	1.89	16.96	−13.21	16.11	−15.40	1.50	3.19	14.46	−23.74	12.36	−30.46
5	4.68	1.81	15.50	−15.52	17.04	−18.15	1.47	3.41	11.07	−23.66	11.68	−27.06
6	3.71	1.83	19.14	−13.13	19.64	−10.91	1.51	3.62	8.24	−33.83	10.67	−26.55
7	3.79	1.87	12.50	−11.05	15.90	−12.94	1.53	3.29	11.13	−24.44	16.38	−21.49
8	4.87	1.82	15.86	−7.25	16.15	−6.54	1.52	3.47	14.10	−25.55	14.94	−26.33
9	5.52	1.83	22.63	−10.86	21.82	−18.09	1.48	3.96	11.47	−27.95	10.90	−29.06
10	3.93	1.85	16.50	−15.82	16.30	−15.34	1.51	3.70	9.79	−20.40	9.89	−20.99
11	8.39	1.86	21.74	−12.84	—	—	1.53	3.46	19.23	−19.66	16.09	−20.25
12	5.98	1.83	24.76	−12.71	—	—	1.49	3.22	12.93	−26.24	12.96	−27.70
13	3.33	1.86	18.10	−14.12	19.01	−13.01	1.46	3.63	14.93	−26.77	14.21	−29.26
14	3.56	1.82	13.02	−17.09	13.13	−17.82	1.57	3.54	12.20	−24.94	11.66	−25.25
15	4.11	1.78	18.09	−3.27	14.92	−8.98	1.51	3.25	13.03	−25.58	13.26	−26.17
16	3.16	1.84	18.59	−5.04	18.64	−6.80	1.51	3.39	10.63	−32.34	7.06	−26.75
17	3.80	1.84	22.59	−6.27	21.55	−8.04	1.54	3.98	13.24	−17.38	12.14	−19.11
18	3.78	1.85	20.17	−6.50	17.40	−15.62	1.48	3.39	14.07	−33.32	14.82	−33.92
19	3.78	1.83	14.69	−15.71	14.88	−16.38	1.50	3.42	11.18	−22.67	10.81	−22.87
20	5.02	1.85	19.68	−6.06	19.47	−7.16	1.54	3.50	9.17	−30.15	7.78	−36.07

**TABLE 5 |:** Mean absolute errors (MAE, kcal mol^−1^) and percent improvement (% Imp.) relative to the dummy model for machine learning algorithms trained on SD–N, SD–CE, and CE–N distances.

	Δ*G*^‡^				Δ*G*_rxn_			
Model	MAE no exp.	MAE exp.	% Imp. no exp.	% Imp. exp.	MAE no exp.	MAE exp	% Imp. no exp.	% Imp. exp.
Elastic net	2.67	2.60	17.63	23.36	4.53	4.49	27.12	36.97
Lasso	2.69	2.68	16.85	20.91	4.65	4.34	25.16	39.10
SVR	2.71	2.79	16.26	17.76	4.31	4.19	30.65	41.12
Ridge	2.83	2.66	12.57	21.66	5.07	4.27	18.31	40.10
Linear	2.85	2.66	12.09	21.62	5.20	4.26	16.35	40.19
KNN	2.91	2.57	10.15	24.32	4.98	3.88	19.74	45.48
Random forest	3.24	2.77	−0.11	18.35	4.69	4.31	24.53	39.49
AdaBoost	3.45	2.81	−6.57	16.99	4.47	4.58	27.99	35.68
Gradient boosting	3.66	2.89	−13.01	14.77	5.13	4.75	17.43	33.26
Decision tree	3.96	2.84	−22.27	16.22	6.15	5.03	0.91	29.43
Dummy	3.24	3.39	0.00	0.00	6.21	7.12	0.00	0.00

*Note:* “No Explicit Water” (No exp.) and “With Explicit Waters” (exp.) refer to QM-derived activation and reaction free energies computed using cluster models without explicit water molecules or with first-shell explicit water molecules, respectively.

**TABLE 6 |:** Mean absolute errors (MAEs, kcal mol^−1^) and percent improvement relative to the dummy model (% Imp.) for machine learning algorithms trained on donor–methyl–acceptor distances (SD–CE, CE–N, SD–N) plus solvent descriptors “Combined” (Comb.) and on solvent-only descriptors “Solvent Only” (Solv).

	Δ*G*^‡^				Δ*G*_rxn_			
Model	MAE comb.	MAE solv.	% Imp. comb.	% Imp. solv.	MAE comb.	MAE solv.	% Imp. comb.	% Imp. solv.
AdaBoost	2.40	2.96	29.11	12.77	4.73	5.44	33.58	23.61
Decision Tree	2.55	3.39	24.73	0.06	4.80	4.96	32.65	30.33
Random Forest	2.58	3.04	23.77	10.45	4.28	4.87	39.97	31.64
Linear	2.59	2.84	23.49	16.32	4.26	8.73	40.19	−22.53
Ridge	2.59	2.78	23.47	17.92	4.27	7.77	40.10	−9.11
Elastic Net	2.60	3.21	23.36	5.45	4.49	5.27	36.97	26.02
Gradient Boosting	2.63	3.12	22.37	7.89	4.88	5.17	31.52	27.42
Lasso	2.68	2.82	20.91	16.91	4.34	5.77	39.10	18.98
KNN	2.69	2.85	20.56	15.87	3.88	4.50	45.48	36.88
SVR	2.76	3.06	18.69	9.66	6.20	5.42	12.89	23.85
Dummy	3.39	3.39	0.00	0.00	7.12	7.12	0.00	0.00

**TABLE 7 |:** Mean absolute errors (MAE, kcal mol^−1^) and percent improvement (% Imp.) relative to the dummy model for machine learning algorithms trained on pairwise distances of active-site residues and on interaction-type descriptors of SAM and glycine.

	Δ*G*^‡^				Δ*G*_rxn_			
Model	MAE pairwise	MAE interaction	% Imp. pairwise	% Imp. interaction	MAE pairwise	MAE interaction	% Imp. pairwise	% Imp. interaction
Elastic net	2.42	2.75	25.30	14.93	4.26	4.35	31.35	29.90
KNN	2.53	2.86	21.83	11.71	4.95	4.52	20.29	27.28
Lasso	2.59	2.69	20.11	16.77	4.41	4.30	28.98	30.83
SVR	2.62	2.93	19.06	9.64	4.10	5.33	33.98	14.15
Ridge	2.69	2.94	16.91	9.34	4.98	4.38	19.90	29.46
AdaBoost	2.74	2.82	15.31	13.00	5.13	4.18	17.37	32.78
Linear	2.80	2.94	13.55	9.19	5.33	4.39	14.18	29.37
Random forest	2.82	2.90	12.93	10.44	4.87	4.66	21.65	25.02
Gradient boosting	3.00	3.10	7.48	4.23	5.14	4.82	17.31	22.39
Decision tree	3.41	3.73	−5.43	−15.35	6.13	6.07	1.36	2.31
Dummy	3.24	3.24	0.00	0.00	6.21	6.21	0.00	0.00

## Data Availability

The data that supports the findings of this study are available in the Supporting Information of this article.
