# Supplementary material for: Computational Investigation of GNMT-Catalyzed Methyl Transfer Reaction: Integrating MD, QM, and ML Approaches
Source: J Comput Chem. Author manuscript; Available in PMC 2026 Jun 29. (PMC13312424; doi:10.1002/jcc.70434)
Supplement: SI-1 [file NIHMS2188582-supplement-SI-1.pdf]

Supporting Information for: Computational Investigation of GNMT-Catalyzed Methyl Transfer Reaction: Integrating MD, QM, and ML Approaches

Jonathan Epih, Anjali Arya, Saghar Gomrok, and Qianyi Cheng*

Department of Chemistry, University of Memphis, Memphis Tennessee 38152, USA.

E-mail: qcheng1@memphis.edu

Contents

1. **Figure S1: RMSD Analysis including the first 16 residues** **S3**

2. **Figure S2: PC1 Distributions Based on Active-Site Heavy-Atom Representations** **S4**

3. **Figure S3: Correlation Between PC1 and Computed Activation Free Energies for Active-Site Heavy-Atom Representation** **S4**

4. **Table S1: Residues and frozen atoms for QM models derived from reactant-1 frames** **S5**

5. **Table S2: Residues and frozen atoms for QM models derived from reactant-2 frames** **S6**

6. Table S3: Residues and frozen atoms for QM models derived from product-1 frames S7

7. Table S4: Residues and frozen atoms for QM models derived from product-2 frames S8

8. Table S5: Model performance (R^2) for donor–methyl–acceptor distance features S8

9. Table S6: Model performance (R^2) for combined distance–solvent and solvent features S8

10. Table S7: Model performance (R^2) for pairwise and interaction-type features S9

11. MD Frames PDB files S10

12. QM-cluster models PDB files S11

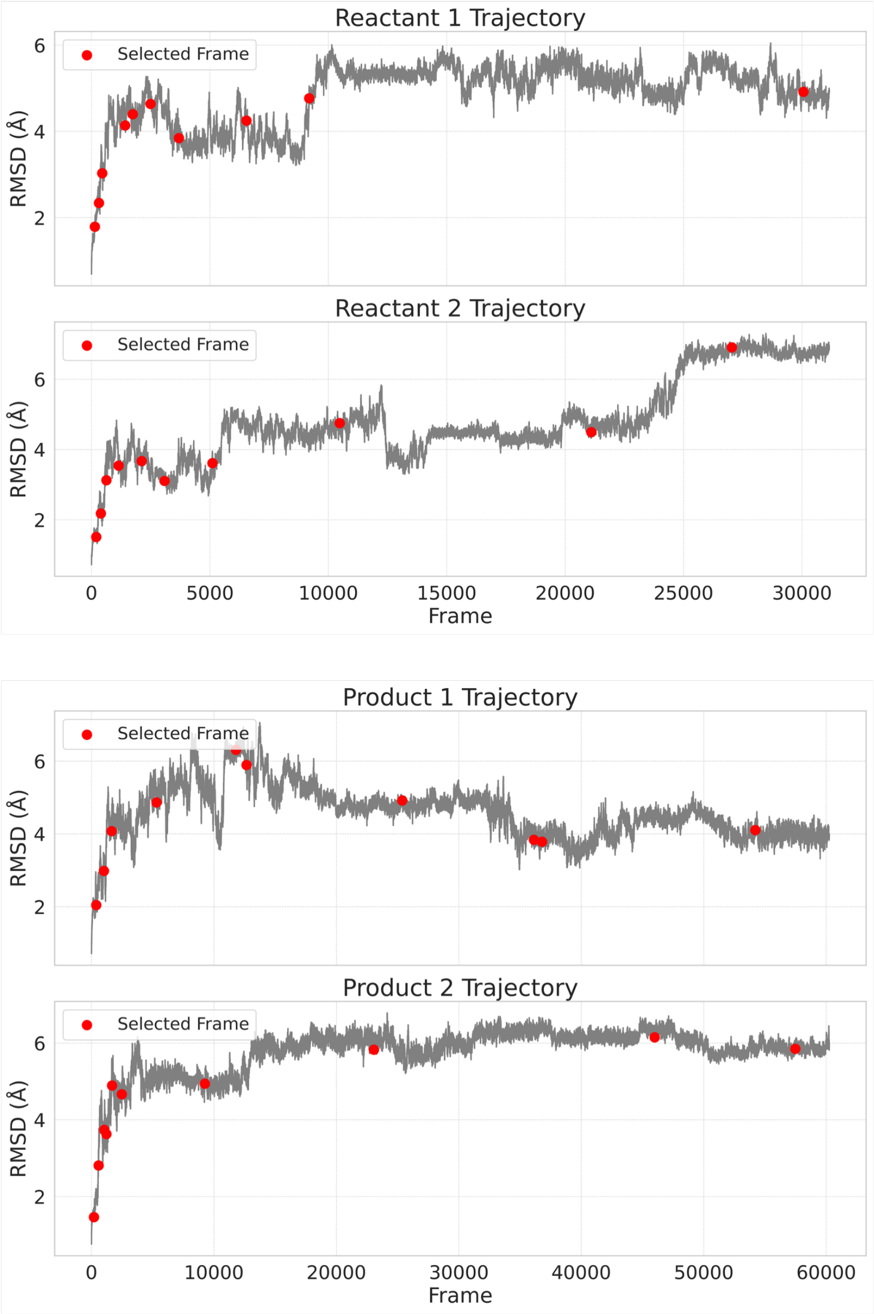

Figure S1: Backbone RMSD (N, C_α, C, and O atoms) relative to the starting structure for each MD trajectory, computed using all residues including the flexible N-terminal 16 residues. Frames selected for QM-cluster modeling are highlighted in red.

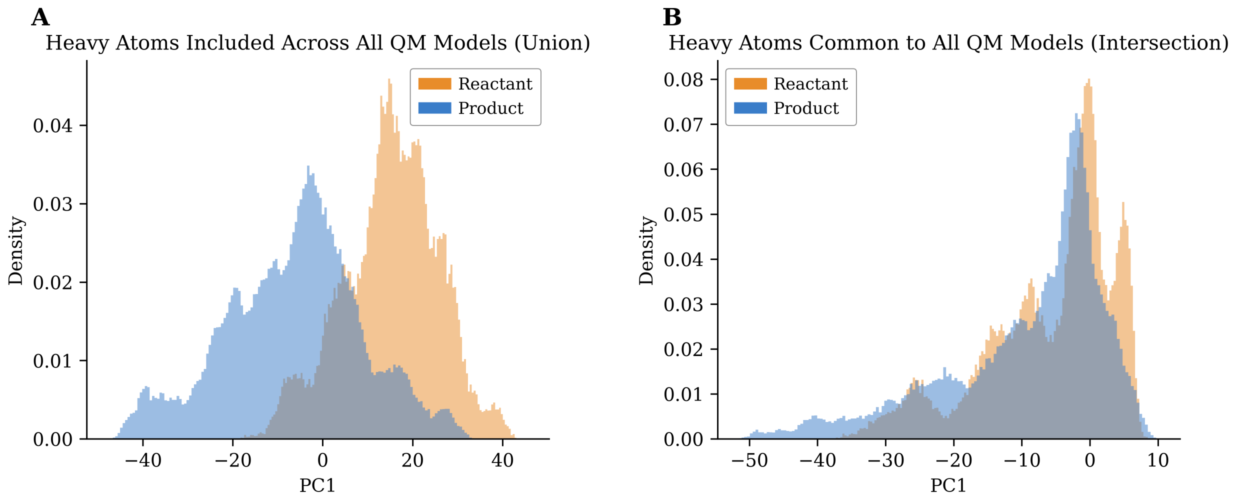

Figure S2: Distribution of PC1 values for reactant-state(orange) and product-state (blue) MD trajectories obtained from PCA performed using active-site heavy atoms under two residue selections.

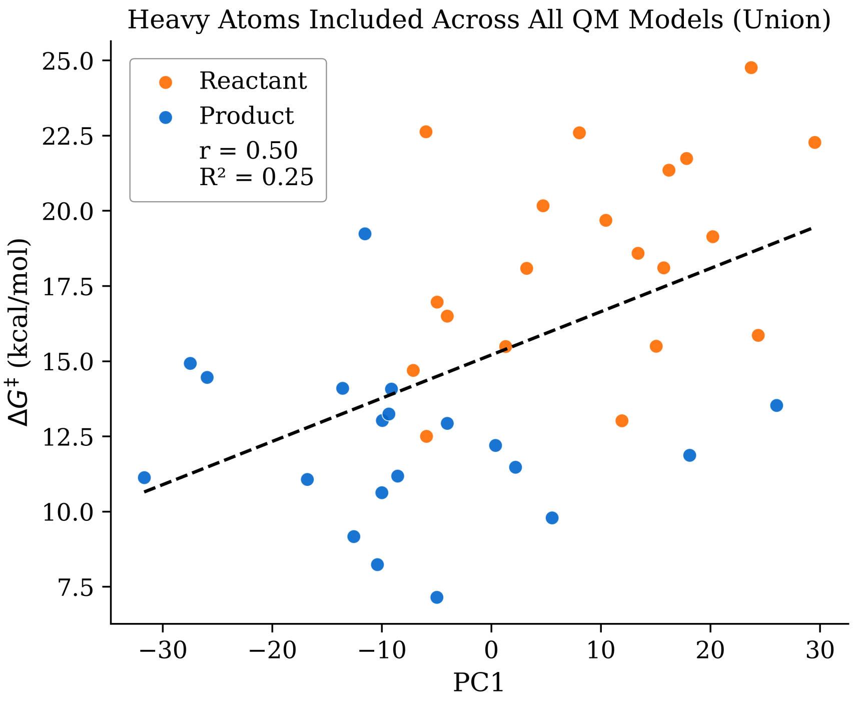

Figure S3: Correlation between PC1 values and QM-computed activation free energies ($\Delta G^{\ddagger}$) for QM-cluster models derived from reactant-state (orange) and product-state (blue) MD simulations. PC1 values were obtained from PCA of the MD trajectories based on the heavy atoms of active-site residues included in all QM-cluster models (union definition).

Table S1: Residue composition, frozen atoms, and QM energy results for the ten QM-cluster models derived from frames selected from the Reactant 1 MD trajectory using hierarchical clustering.

**Residue**	**Frame 17447**	**Frame 3694**	**Frame 30058**	**Frame 1422**	**Frame 2492**	**Frame 9194**	**Frame 141**	**Frame 6544**	**Frame 454**	**Frame 316**
Val12	–	–	C_α C_β	–	–	–	–	–	–	–
Tyr21	C_α C_β	C_α C_β	C_α C_β	C_α C_β	C_α C_β	C_α C_β	C_α C_β	C_α C_β	C_α C_β	C_α C_β
Trp30	C_α C_β	C_α C_β	C_α C_β	C_α C_β	C_α C_β	C_α C_β	C_α C_β	C_α C_β	C_α C_β	C_α C_β
Tyr33	C_α C_β	C_α C_β	C_α C_β	C_α C_β	C_α C_β	C_α C_β	C_α C_β	C_α C_β	C_α C_β	C_α C_β
Arg40	C_α C_β	C_α C_β	C_α C_β	C_α C_β	C_α C_β	C_α C_β	C_α C_β	C_α C_β	C_α C_β	C_α C_β
Ala64	C_α	C_α	C_α	C_α	C_α	C_α	C_α	C_α	C_α	C_α
Cys65	C_α	C_α	C_α	C_α	C_α	C_α	C_α	C_α	C_α	C_α
Gly66	C_α	C_α	C_α	C_α	C_α	C_α	C_α	C_α	C_α	C_α
Thr67	–	–	–	–	C_α	C_α	–	–	–	–
Val69	–	–	–	C_α C_β	–	C_α C_β	–	C_α C_β	–	–
Asp70	C_α C_β	C_α C_β	C_α C_β	C_α C_β	C_α C_β	C_α C_β	C_α C_β	C_α C_β	C_α C_β	C_α C_β
Asp85	C_α C_β	C_α	C_α	C_α	C_α	C_α	C_α C_β	C_α	C_α C_β	C_α
Ala86	C_α	C_α	C_α	C_α	C_α	C_α	C_α	C_α	C_α	C_α
Ser87	C_α	C_α	C_α	C_α	C_α	C_α	C_α	C_α C_β	C_α	C_α
Met90	–	C_α C_β	–	C_α C_β	C_α C_β	C_α C_β	C_α C_β	–	–	–
Ala115	C_α	C_α	C_α	C_α	C_α	C_α	C_α	C_α	C_α	C_α
Asn116	C_α	C_α	C_α	C_α	C_α	C_α	C_α	C_α	C_α	C_α
Trp117	C_α C_β	C_α C_β	C_α C_β	C_α C_β	C_α C_β	C_α C_β	C_α C_β	C_α C_β	C_α C_β	C_α C_β
Cys135	–	–	–	C_α	C_α	C_α	–	–	C_α	–
Leu136	C_α	C_α	C_α	C_α	C_α	C_α	C_α	C_α	C_α	C_α
Gly137	C_α	C_α	C_α	C_α	C_α	C_α	C_α	C_α	C_α	C_α
Asn138	C_α	C_α	C_α	C_α	C_α	C_α	C_α	C_α	C_α	C_α
Ser139	C_α	C_α	C_α	C_α	C_α	C_α	C_α	C_α	C_α	C_α
His142	C_α C_β	C_α C_β	C_α C_β	C_α C_β	C_α C_β	C_α C_β	C_α C_β	C_α C_β	C_α C_β	C_α C_β
Leu143	C_α C_β	C_α C_β	–	C_α C_β	C_α C_β	C_α C_β	–	C_α C_β	C_α C_β	–
Arg175	C_α C_β	C_α C_β	C_α C_β	C_α C_β	C_α C_β	C_α C_β	C_α C_β	C_α C_β	C_α C_β	C_α C_β
Tyr194	C_α C_β	C_α C_β	C_α C_β	C_α C_β	C_α C_β	C_α C_β	C_α C_β	C_α C_β	C_α C_β	C_α C_β
Tyr220	C_α C_β	–	–	–	C_α C_β	C_α C_β	C_α C_β	C_α C_β	C_α C_β	C_α C_β
Leu240	C_α C_β	C_α C_β	C_α C_β	C_α C_β	C_α C_β	C_α C_β	–	C_α C_β	C_α C_β	–
Tyr242	C_α C_β	C_α C_β	C_α C_β	C_α C_β	C_α C_β	C_α C_β	C_α C_β	C_α C_β	C_α C_β	C_α C_β
Tyr283	C_α C_β	C_α C_β	C_α C_β	C_α C_β	C_α C_β	C_α C_β	C_α C_β	C_α C_β	C_α C_β	C_α C_β
**RMSD (Å)**	5.20	3.70	4.89	4.18	4.65	4.62	1.73	4.36	3.02	2.41
**Number of Atoms**	426	438	405	448	458	474	408	445	440	397
$\Delta G^\ddagger$ **(no explicit water)**	22.27	21.35	15.49	16.96	15.50	19.14	12.50	15.86	22.63	16.50
ΔG_{rxn} **(no explicit water)**	2.70	-15.46	-8.28	-13.21	-15.52	-13.13	-11.05	-7.25	-10.86	-15.82
$\Delta G^\ddagger$ **(with explicit water)**	24.70	15.88	16.38	16.11	17.04	19.64	15.90	16.15	21.82	16.30
ΔG_{rxn} **(with explicit water)**	2.40	-12.79	-11.80	-15.40	-18.15	-10.91	-12.94	-6.54	-18.09	-15.34

Table S2: Residue composition, frozen atoms, and QM energy results for the ten QM-cluster models derived from frames selected from the Reactant 2 MD trajectory using hierarchical clustering.

**Residue**	**Frame 21104**	**Frame 27034**	**Frame 10481**	**Frame 5107**	**Frame 3085**	**Frame 2121**	**Frame 1145**	**Frame 394**	**Frame 201**	**Frame 632**
Tyr21	C_α C_β	C_α C_β	C_α C_β	C_α C_β	C_α C_β	C_α C_β	C_α C_β	C_α C_β	C_α C_β	C_α C_β
Trp30	C_α C_β	C_α C_β	C_α C_β	C_α C_β	C_α C_β	C_α C_β	C_α C_β	C_α C_β	C_α C_β	C_α C_β
Tyr33	C_α C_β	C_α C_β	C_α C_β	C_α C_β	C_α C_β	C_α C_β	C_α C_β	C_α C_β	C_α C_β	C_α C_β
Ile34	C_α C_β	–	C_α C_β	–	–	–	C_α C_β	–	–	–
Arg40	C_α C_β	C_α C_β	C_α C_β	C_α C_β	C_α C_β	C_α C_β	C_α C_β	C_α C_β	C_α C_β	C_α C_β
Ala64	C_α	C_α	C_α	C_α	C_α	C_α	C_α	C_α	C_α	C_α
Cys65	C_α	C_α	C_α	C_α	C_α	C_α	C_α	C_α	C_α	C_α
Gly66	C_α	C_α	C_α	C_α	C_α	C_α	C_α	C_α	C_α	C_α
Gly68	–	–	–	–	–	–	–	–	C_α	–
Val69	–	–	–	C_α C_β	C_α C_β	C_α C_β	–	C_α C_β	C_α	C_α C_β
Asp70	C_α C_β	C_α C_β	C_α C_β	C_α C_β	C_α C_β	C_α C_β	C_α C_β	C_α	C_α C_β	C_α C_β
Asp85	C_α	C_α C_β	C_α	C_α	C_α	C_α C_β	C_α	C_α	C_α C_β	C_α C_β
Ala86	C_α	C_α	C_α	C_α	C_α	C_α	C_α	C_α	C_α	C_α
Ser87	C_α C_β	–	C_α C_β	C_α C_β	C_α	C_α	C_α C_β	C_α	C_α	C_α C_β
Met90	C_α C_β	C_α C_β	C_α C_β	C_α C_β	C_α C_β	C_α C_β	C_α C_β	C_α C_β	C_α C_β	C_α C_β
Ala115	C_α	C_α	C_α	C_α	C_α	C_α	C_α	–	–	–
Asn116	C_α	C_α	C_α	C_α	C_α	C_α	C_α	C_α	C_α	C_α
Trp117	C_α C_β	C_α C_β	C_α C_β	C_α C_β	C_α C_β	C_α C_β	C_α C_β	C_α C_β	C_α C_β	C_α C_β
Leu118	–	–	–	–	–	–	–	–	C_α C_β	–
Cys135	–	–	–	–	C_α C_β	C_α C_β	C_α C_β	C_α C_β	–	C_α C_β
Leu136	C_α	C_α	C_α	C_α	C_α	C_α	C_α	C_α	C_α	C_α
Gly137	C_α	C_α	C_α	C_α	C_α	C_α	C_α	C_α	C_α	C_α
Asn138	C_α	C_α	C_α	C_α C_β	C_α	C_α	C_α	C_α	C_α	C_α
Ser139	C_α	C_α	C_α	C_α	C_α	C_α	C_α	C_α	C_α	C_α
His142	C_α C_β	C_α C_β	C_α C_β	C_α C_β	C_α C_β	C_α C_β	C_α C_β	C_α C_β	C_α C_β	C_α C_β
Leu143	–	C_α C_β	–	–	–	–	C_α C_β	–	–	–
Arg175	C_α C_β	C_α C_β	C_α C_β	C_α C_β	C_α C_β	C_α C_β	C_α C_β	C_α C_β	C_α C_β	C_α C_β
Tyr194	C_α C_β	C_α C_β	C_α C_β	C_α C_β	C_α C_β	C_α C_β	C_α C_β	C_α C_β	C_α C_β	C_α C_β
Leu218	C_α C_β	C_α C_β	–	–	–	–	–	–	–	–
Tyr220	C_α C_β	C_α C_β	C_α C_β	C_α C_β	C_α C_β	C_α C_β	C_α C_β	C_α C_β	C_α C_β	C_α C_β
Leu240	–	C_α C_β	–	–	C_α C_β	C_α C_β	C_α C_β	C_α C_β	–	C_α C_β
Tyr242	C_α C_β	C_α C_β	C_α C_β	C_α C_β	C_α C_β	C_α C_β	C_α C_β	C_α C_β	C_α C_β	C_α C_β
Tyr283	C_α C_β	C_α C_β	C_α C_β	C_α C_β	C_α C_β	C_α C_β	C_α C_β	C_α C_β	C_α C_β	C_α C_β
**RMSD (Å)**	4.40	6.82	4.67	3.64	3.13	3.65	3.62	2.14	1.44	3.33
**Number of Atoms**	445	454	428	431	452	452	474	445	447	445
$\Delta G^\ddagger$ **(no explicit water)**	21.74	24.76	18.10	13.02	18.09	18.59	22.95	20.17	14.69	19.68
ΔG_{rxn} **(no explicit water)**	-12.84	-12.71	-14.12	-17.09	-3.27	-5.04	-6.27	-6.50	-15.71	-6.06
$\Delta G^\ddagger$ **(with explicit water)**	–	–	19.01	13.13	14.92	18.64	21.55	17.40	14.88	19.47
ΔG_{rxn} **(with explicit water)**	–	–	-13.01	-17.82	-8.98	-6.80	-8.04	-15.62	-16.38	-7.16

Table S3: Residue composition, frozen atoms, and QM energy results for the ten QM-cluster models derived from frames selected from the Product 1 MD trajectory using hierarchical clustering.

**Residue**	**Frame 54209**	**Frame 25369**	**Frame 5310**	**Frame 36794**	**Frame 11795**	**Frame 12659**	**Frame 36131**	**Frame 385**	**Frame 1646**	**Frame 1016**
Ile17	–	–	–	–	C_α C_β	–	–	–	–	–
Tyr21	C_α C_β	C_α C_β	C_α C_β	C_α C_β	C_α C_β	C_α C_β	C_α C_β	C_α C_β	C_α C_β	C_α C_β
Trp30	C_α C_β	C_α C_β	C_α C_β	C_α C_β	C_α C_β	C_α C_β	C_α C_β	C_α C_β	C_α C_β	C_α C_β
Tyr33	C_α C_β	C_α C_β	C_α C_β	C_α C_β	C_α C_β	C_α C_β	C_α C_β	C_α C_β	C_α C_β	C_α C_β
Ile34	–	–	–	C_α C_β	C_α C_β	–	C_α C_β	C_α C_β	C_α C_β	C_α C_β
Arg40	C_α C_β	C_α C_β	C_α C_β	C_α C_β	C_α C_β	C_α C_β	C_α C_β	C_α C_β	C_α C_β	C_α C_β
Ala64	C_α	C_α	C_α	C_α	C_α	C_α	C_α	C_α	C_α	C_α
Cys65	C_α	C_α	C_α	C_α	C_α	C_α	C_α	C_α	C_α	C_α
Gly66	C_α	C_α	C_α	C_α	C_α	C_α	C_α	C_α	C_α	C_α
Val69	–	–	C_α C_β	C_α C_β	C_α C_β	–	–	–	–	–
Asp70	C_α C_β	C_α C_β	C_α C_β	C_α C_β	C_α C_β	C_α C_β	C_α C_β	C_α C_β	C_α C_β	C_α C_β
Asp85	C_α	C_α C_β	C_α C_β	C_α C_β	C_α C_β	C_α	C_α C_β	C_α	C_α	C_α
Ala86	C_α	C_α	C_α	C_α	C_α	C_α	C_α	C_α	C_α	C_α
Ser87	–	–	C_α C_β	C_α C_β	C_α	C_α	C_α	C_α	–	C_α
Met90	C_α C_β	C_α C_β	C_α C_β	C_α C_β	C_α C_β	C_α C_β	C_α C_β	C_α C_β	–	C_α C_β
Ala115	C_α	C_α	C_α	C_α	–	C_α	C_α	C_α	C_α	C_α
Asn116	C_α	C_α	C_α	C_α	C_α	C_α	C_α	C_α	C_α	C_α
Trp117	C_α C_β	C_α C_β	C_α C_β	C_α C_β	C_α C_β	C_α C_β	C_α C_β	C_α C_β	C_α C_β	C_α C_β
Leu136	C_α	C_α	C_α	C_α	C_α	C_α	C_α	C_α	C_α	C_α
Gly137	C_α	C_α	C_α	C_α	C_α	C_α	C_α	C_α	C_α	C_α
Asn138	C_α	C_α	C_α	C_α	C_α	C_α	C_α	C_α	C_α C_β	C_α
Ser139	C_α C_β	C_α	C_α	C_α	C_α	C_α C_β	C_α	C_α	C_α	C_α
His142	C_α	C_α C_β	C_α C_β	C_α C_β	C_α C_β	C_α C_β	C_α C_β	C_α C_β	C_α C_β	C_α C_β
Leu143	C_α C_β	C_α C_β	–	C_α C_β	–	–	C_α C_β	–	–	–
Arg175	C_α C_β	C_α C_β	C_α C_β	C_α C_β	C_α C_β	C_α C_β	C_α C_β	C_α C_β	C_α C_β	C_α C_β
Tyr193	C_α C_β	C_α C_β	–	–	–	–	–	–	–	–
Tyr194	C_α C_β	C_α C_β	C_α C_β	C_α C_β	C_α C_β	C_α C_β	C_α C_β	C_α C_β	C_α C_β	C_α C_β
Tyr220	–	–	C_α C_β	–	–	–	–	C_α C_β	C_α C_β	C_α C_β
Leu240	–	–	C_α C_β	–	–	–	C_α C_β	C_α C_β	–	–
Tyr242	C_α C_β	C_α C_β	C_α C_β	C_α C_β	C_α C_β	C_α C_β	C_α C_β	C_α C_β	C_α C_β	C_α C_β
**RMSD (Å)**	4.05	4.87	4.72	3.81	6.23	5.79	3.72	2.01	4.13	2.85
**Number of Atoms**	400	400	426	428	416	374	429	421	382	408
$\Delta G^\ddagger$ **(no explicit water)**	13.53	11.87	7.15	14.46	11.07	8.24	11.13	14.10	11.47	9.79
ΔG_{rxn} **(no explicit water)**	-22.33	-23.39	-25.21	-23.74	-23.66	-33.83	-24.44	-25.55	-27.95	-20.40
$\Delta G^\ddagger$ **(with explicit water)**	11.32	9.80	7.90	12.36	11.68	10.67	16.38	14.94	10.90	9.89
ΔG_{rxn} **(with explicit water)**	-24.57	-25.70	-23.32	-30.46	-27.06	-26.55	-21.49	-26.33	-29.06	-20.99

Table S4: Residue composition, frozen atoms, and QM energy results for the ten QM-cluster models derived from frames selected from the Product 2 MD trajectory using hierarchical clustering.

**Residue**	**Frame 45992**	**Frame 23066**	**Frame 57472**	**Frame 9253**	**Frame 2473**	**Frame 1040**	**Frame 203**	**Frame 1679**	**Frame 1221**	**Frame 584**
Ile17	C_α	–	C_α	C_α	C_α	–	–	C_α	C_α	C_α
Pro18	C_α	–	C_α	C_α	C_α	–	–	C_α	C_α	C_α
Asp19	C_α	–	C_α	C_α	C_α	–	–	C_α	C_α	C_α
Tyr21	C_α C_β	C_α C_β	C_α C_β	C_α C_β	C_α C_β	C_α C_β	C_α C_β	C_α C_β	C_α C_β	C_α C_β
Trp30	C_α C_β	C_α C_β	C_α C_β	C_α C_β	C_α C_β	C_α C_β	C_α C_β	C_α C_β	C_α C_β	C_α C_β
Tyr33	–	–	–	–	C_α C_β	C_α C_β	C_α C_β	C_α C_β	C_α C_β	–
Ile34	C_α C_β	C_α C_β	C_α C_β	–	–	–	–	–	C_α C_β	–
Arg40	C_α C_β	C_α C_β	C_α C_β	C_α C_β	C_α C_β	C_α C_β	C_α C_β	C_α C_β	C_α C_β	C_α C_β
Ala64	C_α	C_α	C_α	C_α	C_α	C_α	C_α	C_α	C_α	C_α
Cys65	C_α	C_α	C_α	C_α	C_α	C_α	C_α	C_α	C_α	C_α
Gly66	C_α	C_α	C_α	C_α	C_α	C_α	C_α	C_α	C_α	C_α
Val69	C_α C_β	–	C_α C_β	–	–	–	–	–	–	C_α C_β
Asp70	C_α C_β	C_α C_β	C_α C_β	C_α C_β	C_α C_β	C_α C_β	C_α C_β	C_α C_β	C_α C_β	C_α C_β
Val84	–	–	–	–	C_α C_β	–	–	–	–	–
Asp85	C_α C_β	C_α	C_α C_β	C_α C_β	C_α	C_α C_β	C_α	C_α C_β	C_α	C_α
Ala86	C_α	C_α	C_α	C_α	C_α	C_α	C_α	C_α	C_α	C_α
Ser87	C_α C_β	C_α C_β	C_α	C_α	C_α	C_α	C_α	–	C_α C_β	–
Met90	C_α C_β	C_α C_β	C_α C_β	–	C_α C_β	–	C_α C_β	–	C_α C_β	–
Ala115	C_α	C_α	C_α	C_α	C_α	C_α	C_α	C_α	C_α	C_α
Asn116	C_α	C_α	C_α	C_α	C_α	C_α	C_α	C_α	C_α	C_α
Trp117	C_α C_β	C_α C_β	C_α C_β	C_α C_β	C_α C_β	C_α C_β	C_α C_β	C_α C_β	C_α C_β	C_α C_β
Leu118	–	–	–	–	C_α C_β	–	–	–	–	–
Leu136	C_α	C_α	C_α	C_α	C_α	C_α	C_α	C_α	C_α	C_α
Gly137	C_α	C_α	C_α	C_α	C_α	C_α	C_α	C_α	C_α	C_α
Asn138	C_α	C_α	C_α	C_α C_β	C_α C_β	C_α C_β	C_α	C_α C_β	C_α C_β	C_α C_β
Ser139	C_α	C_α	C_α C_β	C_α	C_α	C_α	C_α	C_α	–	C_α
Phe140	–	–	–	C_α	C_α	C_α	C_α	C_α	–	C_α
His142	C_α C_β	C_α C_β	C_α C_β	C_α C_β	C_α C_β	C_α C_β	C_α C_β	C_α C_β	C_α C_β	C_α C_β
Leu143	–	–	–	C_α C_β	C_α C_β	–	–	C_α	C_α C_β	C_α C_β
Arg175	C_α C_β	C_α C_β	C_α C_β	C_α C_β	C_α C_β	C_α C_β	C_α C_β	C_α C_β	C_α C_β	C_α C_β
Tyr194	C_α C_β	C_α C_β	C_α C_β	C_α C_β	C_α C_β	C_α C_β	C_α C_β	C_α C_β	C_α C_β	C_α C_β
Tyr220	–	–	–	C_α C_β	C_α C_β	C_α C_β	C_α C_β	C_α C_β	C_α C_β	–
Leu240	C_α C_β	C_α C_β	C_α C_β	C_α C_β	C_α C_β	–	–	C_α C_β	C_α C_β	C_α C_β
Tyr242	C_α C_β	C_α C_β	C_α C_β	C_α C_β	C_α C_β	C_α C_β	C_α C_β	C_α C_β	C_α C_β	C_α C_β
Tyr283	C_α C_β	C_α C_β	C_α C_β	C_α C_β	–	–	–	–	–	C_α C_β
**RMSD (Å)**	6.24	5.85	5.80	4.95	4.56	3.63	1.37	4.87	3.48	2.82
**Number of Atoms**	447	408	447	443	482	377	400	436	456	429
$\Delta G^\ddagger$ **(no explicit water)**	19.23	12.93	14.93	12.20	13.03	10.63	13.24	14.07	11.18	9.17
ΔG_{rxn} **(no explicit water)**	-19.66	-26.24	-26.77	-24.94	-25.58	-32.34	-17.38	-33.32	-22.67	-30.15
$\Delta G^\ddagger$ **(with explicit water)**	16.09	12.96	14.21	11.66	13.26	7.06	12.14	14.82	10.81	7.78
ΔG_{rxn} **(with explicit water)**	-20.25	-27.70	-29.26	-25.25	-26.17	-26.75	-19.11	-33.92	-22.87	-36.07

Table S5: Coefficient of determination (R^2) values for machine learning algorithms trained on SD–N, SD–CE, and CE–N distances. "No Explicit Water" (No Exp.) and "With Explicit Waters" (Exp.) denote models trained using features derived from QM cluster calculations without explicit water molecules and with first-shell explicit water molecules, respectively.

	$\Delta G^\ddagger$		ΔG_{rxn}	
**Model**	$\mathbf{R^2}$ **No Exp.**	$\mathbf{R^2}$ **Exp.**	$\mathbf{R^2}$ **No Exp.**	$\mathbf{R^2}$ **Exp.**
Support Vector Regressor	0.22	0.32	0.43	0.53
Elastic Net Regression	0.20	0.40	0.39	0.51
Lasso Regression	0.20	0.37	0.35	0.52
KNN Regressor	0.18	0.41	0.27	0.56
Ridge Regression	0.12	0.38	0.23	0.50
Linear Regression	0.11	0.38	0.19	0.50
Random Forest Regressor	-0.06	0.39	0.34	0.45
AdaBoost Regressor	-0.14	0.36	0.40	0.34
Gradient Boosting Regressor	-0.27	0.30	0.21	0.38
Decision Tree Regressor	-0.40	0.39	-0.20	0.25

Table S6: Coefficient of determination (R^2) values for machine learning algorithms trained on donor–methyl–acceptor distances (SD–CE, CE–N, SD–N) combined with solvent descriptors ("Combined", Comb.) and on solvent-only descriptors ("Solvent Only", Solv).

**Model**	Comb.		Solv.	
	$\mathbf{R}^2$ $\Delta G^\ddagger$	$\mathbf{R}^2$ ΔG_{rxn}	$\mathbf{R}^2$ $\Delta G^\ddagger$	$\mathbf{R}^2$ ΔG_{rxn}
AdaBoost Regressor	0.51	0.33	-0.08	0.15
Decision Tree Regressor	0.42	0.29	-0.30	0.29
Random Forest Regressor	0.44	0.44	-0.14	0.35
Ridge Regression	0.41	0.50	0.05	-0.70
Linear Regression	0.39	0.50	0.03	-1.35
Elastic Net Regression	0.40	0.51	0.05	0.30
Gradient Boosting Regressor	0.40	0.34	-0.21	0.24
Lasso Regression	0.37	0.52	0.14	0.11
KNN Regressor	0.33	0.56	0.03	0.39
Support Vector Regressor	0.27	0.18	-0.04	0.19

Table S7: Coefficient of determination (R^2) values for machine learning algorithms trained on pairwise distances of active-site residues and on interaction-type descriptors of SAM and glycine.

**Model**	Pairwise		Interaction	
	$\mathbf{R}^2$ $\Delta G^\ddagger$	$\mathbf{R}^2$ ΔG_{rxn}	$\mathbf{R}^2$ $\Delta G^\ddagger$	$\mathbf{R}^2$ ΔG_{rxn}
Elastic Net Regression	0.24	0.43	0.21	0.45
KNN Regressor	0.23	0.28	0.17	0.43
Lasso Regression	0.23	0.38	0.20	0.45
Support Vector Regressor	0.22	0.46	0.11	0.24
Ridge Regression	0.11	0.22	0.14	0.44
AdaBoost Regressor	0.14	0.32	0.13	0.44
Linear Regression	0.05	0.12	0.14	0.44
Random Forest Regressor	0.10	0.26	0.10	0.38
Gradient Boosting Regressor	0.07	0.20	0.05	0.29
Decision Tree Regressor	-0.15	0.07	-0.32	-0.06

MD Frames PDB Files

The PDB files corresponding to the MD frames selected using the early-stage, clustering, and random sampling methods are located in the directory: `MD-frame-pdbs`.

Naming convention: Each PDB file is named according to the selection method and the associated MD frame. For clustering and random sampling, filenames follow the pattern: `<method>-<state>-<frame>.pdb`, e.g., `clustering-P1-385.pdb`, where:

- `early_stage`, `clustering`, and `sampling` denote the selection method,
- `R1`, `R2`, `P1`, and `P2` indicate the reactant-1, reactant-2, product-1, and product-2 trajectories, respectively,
- the numeric suffix corresponds to the MD frame number.

Early-stage frames do not use MD frame numbers; instead, they are labeled `Frame_A`, `Frame_B`, `Frame_C`, and `Frame_D`.

Note: The residue labeled `ACT 294` in the PDB files corresponds to the glycine substrate.

QM-cluster models PDB files

The PDB files corresponding to the optimized reactant, transition-state (TS), and product structures generated from the early-stage, clustering, and randomly sampled MD frames are located in the directory: `QM-cluster-pdbs`.

Naming convention: QM-cluster model PDB files follow the pattern: `method_frame_state_solvation.pdb`, e.g., `clustering_141_R1_w_p.pdb`, where:

- `early_stage`, `clustering`, and `sampling` denote the selection method.
- the frame identifier matches the corresponding MD-frame label from which the cluster was built.
- `R1`, `R2`, `P1`, or `P2` denote the simulation state.
- `w` or `wo` indicate whether explicit water molecules were included or not included, respectively.
- `r`, `ts`, and `p` specify the optimized reactant, transition-state, or product structure.

Note: The residue labeled `ACT 294` in the PDB files corresponds to the glycine substrate.
